# Integration of chromosome conformation and gene expression networks reveals regulatory mechanisms in triple negative breast cancer

**DOI:** 10.3389/fcell.2025.1597245

**Published:** 2025-07-04

**Authors:** Helena Reyes-Gopar, Keila Adonai Pérez-Fuentes, Matthew L. Bendall, Enrique Hernández-Lemus

**Affiliations:** ^1^ Programa de Doctorado en Ciencias Biomédicas, Universidad Nacional Autónoma de México, Mexico City, Mexico; ^2^ Feinstein Institutes for Medical Research, Northwell Health, Institute of Translational Research, Manhasset, NY, United States; ^3^ Computational Genomics Division, National Institute of Genomic Medicine, Mexico City, Mexico; ^4^ Weill Cornell Medicine, Division of Infectious Diseases, New York, NY, United States; ^5^ Centro de Ciencias de la Complejidad, Universidad Nacional Autónoma de México, Mexico City, Mexico

**Keywords:** triple negative breast cancer, chromosome conformation capture, hi-c, complex networks, regulatory genomics

## Abstract

**Introduction:**

Triple-negative breast cancer (TNBC) accounts for twelve percent of all breast cancer cases, with a survival rate around ten percent lower than ER+/PR+ positive breast cancers. There are limited therapeutic options as these tumors do not respond to hormonal therapy or HER2-targeted treatments. We hypothesized that new insights into pathogenic mechanisms in TNBC can be obtained from studying epigenetic alterations through Hi-C (genome-wide chromosome conformation capture) data analysis.

**Methods:**

We developed a computational strategy that captured key properties of chromatin conformation while incorporating statistical measures of interaction significance. This model addresses limitations in Hi-C data analysis without relying on predefined features like TADs and compartments. We applied this model to Hi-C and RNA-seq data from TNBC patients, representing the data as multilayer networks to identify genome-wide properties of the TNBC 3D genome.

**Results:**

Our network-based analysis revealed distinct chromatin interaction patterns in TNBC compared to healthy contralateral controls. Hi-C data can distinguish interaction patterns related to diseased phenotypes or interaction patterns with potential to exert regulatory effects instead of incidental contacts, but some apparently random interactions may also support important genome regulatory activities.

**Discussion:**

Our findings demonstrate that network-based Hi-C analysis can capture the genome-wide complexity of chromatin interactions in TNBC. This integrative approach provides new insights into the epigenetic mechanisms underlying TNBC pathogenesis and contributes to the advancement of analysis methods for future investigations into novel therapeutic targets.

## 1 Introduction

Triple-negative breast cancer (TNBC), characterized by the absence of estrogen (ER) and progesterone (PR) receptors, and HER2 amplification, is one of the most challenging subtypes of breast cancer. TNBC accounts for 12% of all breast cancer cases, with a higher prevalence observed in younger women. Despite advancements in early detection and treatment, the 5-year survival rate for TNBC remains 8%–16% lower than for ER+/PR + positive breast cancers (Howard and Olopade, n.d.). Currently, there are limited therapeutic options to target the molecular profile of TNBC, as these tumors do not respond to hormonal therapy or HER2-targeted treatments.

TNBC is characterized by a rapid proliferation rate, high histological grade (Dent et al., 2007), and often affects younger patients (Malorni et al., 2012). Compared to other breast cancer subtypes, TNBC has a greater propensity to metastasize, particularly to visceral organs such as the brain, liver, and lungs (Xiao et al., 2018). The absence of hormone receptors and HER2 amplification complicates treatment of TNBC, as targeted therapies remain unavailable for this subtype. These factors collectively contribute to the poor prognosis and survival (Carey et al., 2006) associated with TNBC, highlighting the urgent need for novel approaches to better understand its underlying biology and develop more effective therapeutic strategies (Bianchini et al., 2022).

The epigenetic characterization of diseases at the molecular level has become increasingly important for understanding pathogenic mechanisms. Epigenetic alterations, including chromatin remodeling, can impact transcriptional programs and contribute to cancer initiation, progression, and maintenance. Hi-C is a genome-wide chromosome conformation capture technique that identifies physical interactions between genomic regions by crosslinking chromatin, digesting DNA, ligating spatially proximal fragments, and sequencing the resulting junctions to map chromosomal contacts. These interactions enable spatial and temporal regulation of gene expression, and their disruption has been implicated in various disease phenotypes (Lupiáñez et al., 2015; Martin et al., 2015), the activation of protoncogenes (Hnisz et al., 2016), and enhancer hijacking (Weischenfeldt et al., 2017). However, identifying biologically meaningful chromatin interactions remains a significant challenge. DNA, as a long polymer confined within the nucleus, naturally exhibits numerous random interactions. Therefore, a fundamental task is to analyze Hi-C data distinguishing interaction patterns related to diseased phenotypes or interaction patterns with potential to exert regulatory effects instead of incidental contacts, while simultaneously recognizing that some apparently random interactions may support important genome regulatory activities.

Hi-C analyses, to identify chromatin interactions in cancer phenotypes, traditionally rely on strategies such as observed/expected methods and iterative correction matrix balancing and ultimately detecting predetermined structural features such as Topologically Associating Domains (TAD) and A/B compartments (Peng et al., 2022; Zhang et al., 2024). While these approaches have successfully identified important organizational principles of the genome and incorporate key factors, including the distance-decay effect, correcting for technical biases inherited from the experimental procedures, and the equal visibility principle, they may not capture the genome-wide complexity of chromatin interactions.

In this work, we analyzed publicly available Hi-C data from TNBC tumors by developing a computational model that captures key properties of chromatin conformation while incorporating statistical measures of interaction significance. By representing the data as networks, we identified genome-wide properties of the TNBC 3D genome. While several groups have utilized network representations of chromosome conformation capture data primarily for 3D molecular-polymer modeling, Pancaldi and colleagues pioneered the application of network approaches to regulatory genomics (Pancaldi et al., 2016a; Madrid-Mencía et al., 2020; Malod-Dognin et al., 2020; Pancaldi, 2021), demonstrating how promoter chromosome conformation data could be represented as networks to study regulatory interactions (Pancaldi, 2023). Building on this foundational work, our approach extends these network-based concepts to genome-wide Hi-C data for comprehensive regulatory analysis. Following Pancaldi’s insight that Hi-C data inherently represents a physical interaction network, we leverage this property to enable natural integration with inferred networks from other -omics data such as RNA-seq. In this study, we integrated these networks with transcriptional data to examine how chromatin organization relates to gene expression, demonstrating the power of network-based Hi-C analysis to perform integrative analysis and advance our understanding of genomic regulation across different biological contexts.

## 2 Materials and methods

### 2.1 Hi-C data collection and processing

#### 2.1.1 Human genome reference

We utilized the GRCh38 reference genome and Gencode V36 annotation (https://gdc.cancer.gov/about-data/gdc-data-processing/gdc-reference-files) to ensure compatibility with TCGA Breast Cancer RNA-seq datasets. The reference genome was digested *in silico* using HiC-Pro’s digest_genome.py script at MboI and HindIII restriction sites, corresponding to the Arima protocol. Chromosome sizes were obtained from the UCSC Genome Browser (GRCh38 assembly).

#### 2.1.2 Hi-C data analysis

Raw sequencing reads (FASTQ files) were obtained from GEO (accession GSE167150) and processed through HiC-Pro (Servant et al., 2015), which performs independent alignment of read pairs to the reference genome using bowtie2, quality filtering to remove i) reads with low mapping quality, ii) PCR duplicates, and iii) ligation artifacts such as self-ligations and dangling-end reads, valid interaction assessment, and construction of raw contact matrices.

We assessed replicate correlation between each phenotype’s three raw matrices using hicCorrelate (HiCExplorer, Ramírez et al., 2018). After confirming high correlation (Supplementary Figures S1A, B), we merged the three paired TNBC and three contralateral healthy tissue samples at the deduplicated valid pairs stage. The genome was binned at 40 kb resolution for subsequent analyses.

#### 2.1.3 Hi-C data normalization

Hi-C raw data was normalized to address multiple potential biases in Hi-C data using the Matrix Balancing approach. Iterative correction and eigen vector decomposition (ICE) was employed to correct for known biases including GC content variation, mappability differences, restriction fragment length, and other systematic biases. The ICE method assumes all genomic regions should have equal “visibility”, iteratively balancing the matrix until row and column sums converge. HiCExplorer tools were used to calculate and plot log2 fold change Hi-C matrices and Hi-C frequency distance decay.

#### 2.1.4 Hi-C intrachromosomal networks

We used the non-central hypergeometric distribution model through the HiEdge implementation (Paulsen et al., 2014; Stav, 2024) to identify significant interactions while accounting for the distance-dependent decay of interaction frequencies. For intrachromosomal interactions, HiEdge accounts for the distance-dependent decay of interaction frequency by fitting a monotonically decreasing spline that serves as a null model for significance testing. The probability of observing n_ij_ interactions between loci *i* and *j* is given by:

$$P\left(n_{ij}|n,n_{i},n_{j},\omega_{ij}\right)=\frac{\left(\begin{array}{l} n_{j}\\ n_{ij} \end{array}\right)\left(\begin{array}{l} 2n-n_{i}\\ n_{j}-n_{ij} \end{array}\right)\omega_{ij}^{n_{ij}}}{\sum_{n_{ij}^{\prime }}\left(\begin{array}{l} n_{j}\\ n_{ij}^{\prime } \end{array}\right)\left(\begin{array}{l} 2n-n_{i}\\ n_{j}-n_{ij}^{\prime } \end{array}\right)\omega_{ij}^{n_{ij}^{\prime }}}$$

Where:

*n* is the total interaction count

*n*_*i*_ and *n*_*j*_ are individual loci contact frequencies

*ω*_*ij*_ is the distance-dependent odds ratio.

This approach is particularly suitable for Hi-C data because it models distance-dependent decay explicitly, it accounts for both local and global interaction patterns, and it provides robust statistical significance estimates.

We identified significant intrachromosomal interactions from 40 kb resolution Hi-C data (processed with HiC-Pro) using HiEdge. We removed interactions involving GRCh38 blacklisted regions (https://www.encodeproject.org/files/ENCFF356LFX/), centromeres (https://hgdownload.soe.ucsc.edu/goldenPath/hg38/database/cytoBand.txt.gz), and self-interactions. We used HiC-Pro bias files to correct for expected interaction frequencies (lower bound 0.5, upper bound 2) and fitted the monotonically decreasing spline to the interaction frequency decay with genomic distance using 200-bin metabins to generate the null model. We used a false discovery rate threshold of 0.05 to correct for multiple testing the binomial survival test p-values.

We retained Hi-C interactions with a q-value <0.001 from the 40 kb binned Hi-C matrices for both Normal and Triple-Negative Breast Cancer (TNBC) phenotypes and constructed igraph objects to represent the Normal and TNBC Hi-C intrachromosomal networks for all human chromosomes (chr1-chr22, chrX) by reading the edge lists into R (Csardi et al., 2025). We represented genomic regions (40 kb bins) as nodes and chromatin interactions between them as edges. For each chromosome, we generated an undirected, unweighted graph where edges correspond to significant chromatin interactions.

We included bin genomic coordinates (start, end, midpoint) as node attributes. We assigned GENCODE (v36) annotated genes to nodes containing their transcription start sites (TSS) using the GenomicRanges package (Lawrence et al., 2013). We classified nodes into three categories based on their gene content: coding genes (C), non-coding RNAs (R), or no annotated features (N), and stored this information in the “node_type” attribute.

We included several edge attributes: q-value (corrected p-value of interaction significance), genomic distance in base pairs, Hi-C interaction count, Hi-C count Z-score, and “edge_type” (defined by the node types of the interacting regions, e.g., “C-C″ for interactions between coding regions).

#### 2.1.5 Hi-C interactions Z-Score

We calculated Z-scores for Hi-C interaction counts on a chromosome-by-chromosome basis to account for chromosome-specific interaction patterns. For each chromosome, we subtracted the mean interaction count from each individual count value and divided by the standard deviation of counts within that chromosome

$$Z=\frac{x-\mu }{\sigma }$$

This chromosome-specific normalization allowed us to identify statistically significant interactions while accounting for differences in chromosome size, gene density, and overall chromatin structure.

We stored the processed networks as R objects for subsequent analysis, with separate network objects for each chromosome and phenotype. This approach facilitated chromosome-specific analyses while maintaining the ability to perform cross-chromosome comparisons.

### 2.2 Network structure analysis

#### 2.2.1 Jaccard index

The Jaccard index was used to quantify the similarity between normal and TNBC networks. For nodes, the Jaccard index was calculated as the ratio of the number of common nodes to the total number of unique nodes across both networks (|A∩B|/|A∪B|). Similarly, for edges, the Jaccard index was determined by comparing the edge sets between networks, with edges identified by their endpoint node pairs. This measure provided an objective assessment of topological similarity between the normal and TNBC chromosome-specific networks.

#### 2.2.2 Degree calculation

Network connectivity was assessed through degree calculations for each node in both normal and TNBC networks. The degree of a node represents the number of direct connections (edges) it maintains with other nodes. For each chromosome-specific network, we calculated the degree distribution, average degree, and identified hub nodes (those with significantly higher degrees). These metrics were instrumental in understanding the differences in connectivity patterns between normal and TNBC networks.

#### 2.2.3 Z-weighted degree

To account for the significance of connections, we implemented a Z-weighted degree metric that incorporated edge Z-scores. For each node, the Z-weighted degree was calculated as the sum of the absolute Z-scores of all its connected edges. This provided a more nuanced measure of node importance by considering both the quantity and the statistical significance of connections. The Z-score values represent the strength of correlation between genomic regions, with higher absolute values indicating stronger associations in the chromatin interaction network.

#### 2.2.4 Chromatin interaction profile comparison

To identify regions of the genome that preserve their three-dimensional architecture in TNBC, we implemented a Jaccard dissimilarity analysis comparing chromatin interaction profiles between normal and TNBC samples. For each genomic node, we calculated the Jaccard dissimilarity index between the sets of interactions in normal and tumor samples. Lower dissimilarity values indicate higher preservation of chromatin interactions.

We ranked nodes based on their dissimilarity scores and determined an optimal threshold for selecting highly preserved nodes using the kneedle elbow point detection algorithm. This approach identifies the point of maximum curvature in the ranked dissimilarity plot, achieving an optimal balance between stringency and inclusivity in node selection.

#### 2.2.5 Gene Ontology enrichment analysis

We extracted genes located within the preserved chromatin interaction nodes (dissimilarity ≤0.489) for functional enrichment analysis. To facilitate this process, we converted gene symbols to Entrez IDs using the org.Hs.e.g.,.db annotation package. We then performed Gene Ontology (GO) enrichment analysis using the clusterProfiler R package (Yu et al., 2012), focusing on biological processes (BP) ontology. We calculated enrichment using a hypergeometric test with Benjamini–Hochberg correction for multiple testing, applying a significance threshold of adjusted p-value <0.05 and q-value <0.1.

We used the enrichplot R package to visualize the enriched GO terms. Specifically, we employed the cnetplot function to generate a network representation where edges connect GO terms that share gene annotations, highlighting functional clusters and relationships between biological processes. The resulting network visualization illustrates the functional relationships between GO terms and their associated genes, with node size proportional to statistical significance (p-value).

#### 2.2.6 RNA-seq data collection and preprocessing

RNA-seq data were obtained from The Cancer Genome Atlas (TCGA), comprising 197 Primary Tumor (basal breast cancer) samples and 112 Normal Breast (adjacent tissue) samples. The raw counts matrix initially contained 23,258 genes. Low-expression genes were filtered out using the criterion of ≤10 reads in >80% of samples. Data normalization and batch effect correction were performed using DESeq2 (Love et al., 2014).

#### 2.2.7 Differential gene expression

Raw counts matrices were filtered to exclude lowly expressed genes (<10 reads), size factor normalization based on median ratio normalization between samples was applied and differential gene expression was identified comparing TNBC samples to Normal samples.

We identified differentially expressed genes using DESeq2 on normalized, corrected, and filtered counts matrices. We considered genes differentially expressed if they had a log2 fold change >2 or < −2 and an adjusted p value <0.05.

### 2.3 RNA-seq intrachromosomal coexpression networks

#### 2.3.1 Mutual information calculation and MI threshold

Gene regulatory networks were inferred from the TCGA RNA-seq data using ARACNe (Algorithm for the Reconstruction of Accurate Cellular Networks) (Lachmann et al., 2016). In these networks, genes were represented as nodes, with edges between them indicating mutual information, which quantifies the statistical dependence or shared information between gene expression levels. These networks provided insights into the dynamics of gene expression changes between normal breast tissue and basal breast cancer.

The inference of gene regulatory networks using ARACNE-AP required two primary inputs: a gene expression matrix and a list of regulators. For each chromosome, the corresponding expression matrix derived from the TCGA samples was used, while the list of regulators consisted of the set of expressed genes for each chromosome in each phenotype (normal breast and basal breast cancer). To optimize processing, network inference was executed in parallel, allowing for simultaneous construction of multiple networks. The process was conducted in three key stages:

1. Estimation of the mutual information (MI) threshold: A significance threshold for MI values was determined based on the TCGA gene expression data, using a p-value of 1E-8 as the statistical criterion.
2. Network reconstruction via bootstrapping: A total of 100 MI networks were inferred from random resampling of gene expression profiles from the 197 tumor and 112 normal samples.
3. Consensus network construction: From the 100 generated networks, a final network was obtained by considering the frequency with which each interaction appeared across the bootstrapped networks. The statistical significance of these interactions was assessed using a Poisson distribution, and only those surpassing a significance threshold (P < 0.05, Bonferroni correction) were retained.

#### 2.3.2 Multilayer network construction and analysis

We employed a multilayer network approach to integrate chromatin interaction data (Hi-C) with gene expression correlations to investigate the complex interplay between genomic architecture and gene regulation in normal breast tissue and triple-negative breast cancer (TNBC).

#### 2.3.3 Data integration and network construction

Two primary data types were integrated: (1) Hi-C interaction networks representing three-dimensional chromatin organization at 40 kb resolution, and (2) gene co-expression networks derived from mutual information (MI) calculations using the ARACNE algorithm on TCGA expression data.

Our multilayer networks consisted of two distinct layers: Hi-C layer: Nodes represent genomic regions (40 kb bins), and edges represent significant chromatin interactions between these regions, MI layer: Nodes represent individual genes, and edges represent significant mutual information values between gene pairs, indicating co-expression relationships.

To establish connections between the two layers, we mapped genes from the MI layer to their corresponding genomic regions in the Hi-C layer using the GenomicRanges framework. Each gene in the MI layer was connected to its corresponding Hi-C bin through interlayer edges.

#### 2.3.4 Community detection and comparison

We applied the Louvain community detection algorithm to identify functional modules in the multilayer networks. We implemented the analysis using the cluster_louvain function from the igraph package, which optimizes modularity to find communities in the network. Communities were detected separately for normal and TNBC conditions on a unified graph that combined Hi-C edges (chromatin interactions), MI edges (gene co-expression), and interlayer edges (gene-region mapping).

Edge weights were preserved during community detection, with: Hi-C layer weights representing interaction strength between genomic regions. MI layer weights representing mutual information strength between gene pairs. Interlayer weights set to a default value of 1 to indicate the presence of a connection.

For visualization, we used the Fruchterman-Reingold algorithm to generate network layouts that emphasize community structure. Nodes were shaped according to their layer (squares for Hi-C regions, circles for genes) and colored by community membership. Edges were colored according to their layer (red for Hi-C, blue for MI, gray for interlayer connections).

To quantify changes in community structure between normal and TNBC conditions, we extracted community assignments for each node in both conditions, identified nodes present in both conditions, calculated the percentage of nodes that changed community membership, performed this analysis for all nodes combined and separately for each layer (Hi-C and MI).

## 3 Results

### 3.1 Development of the HiC network analysis pipeline

**FIGURE 1:**
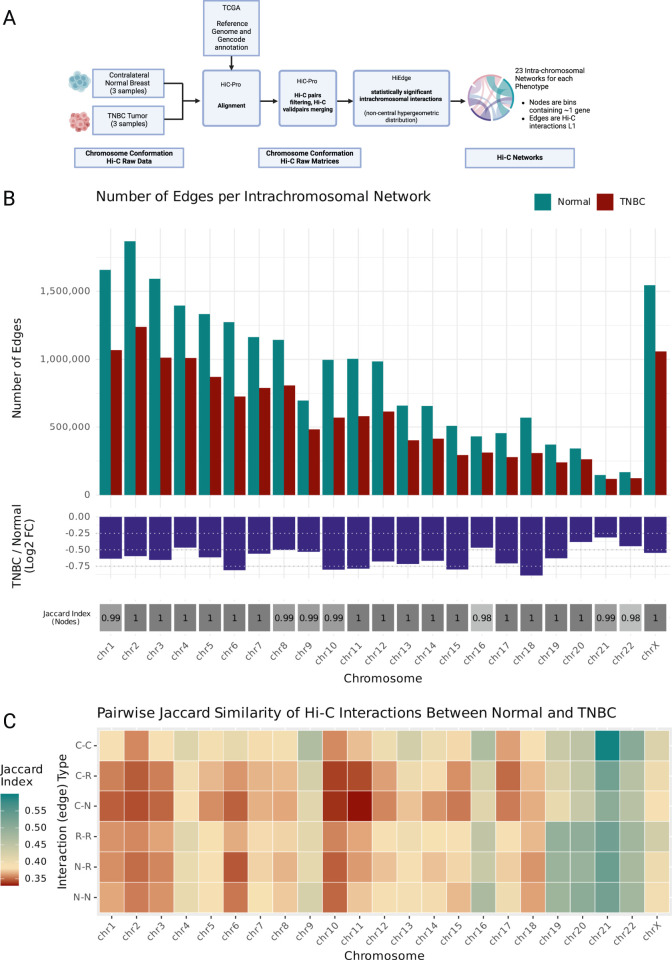
**(A)** Analysis workflow diagram. Hi-C raw data from Triple Negative Breast Cancer tumors (N = 3) and contralateral healthy breast samples was obtained from GEO and aligned to the same reference genome and Gencode annotation than TCGA breast cancer harmonized data, the alignments were processed with HiC-Pro. Hi-C raw interaction matrices were processed using the noncentral hypergeometric distribution in HiEdge to obtain matrices (edge lists) of significant chromatin contacts. The threshold for significant interactions was set at qvalue <0.001. This resulted in one Normal and one TNBC intrachromosomal interactions network for each human chromosome. **(B)** Upper panel: barplots of number of Normal and TNBC edges grouped by chromosome. Middle panel: log fold change of number of TNBC edges relative to Normal edges for each chromosome. Lower panel: Jaccard index for each chromosome’s node set. **(C)** Heatmap of Jaccard index for each chromosome’s edge type set. Edges are classified by Interaction type according to the node’s they are connecting (C: Coding Gene Node, R: ncRNA Node, N: noncoding DNA Node). Created in BioRender. Nixon, D. (2025) https://BioRender.com/lo4eeew/.

We analyzed Hi-C data from Triple Negative Breast Cancer tumors (N = 3), and contralateral healthy breast samples (N = 3) obtained from three patients. Raw sequencing reads were aligned to the CRCh38 reference genome using GENCODE V36 annotation (Figure 1A). The aligned reads were processed using HiC-Pro (Servant et al., 2015) to generate interaction matrices at 40 kb resolution. To identify statistically significant chromatin interactions, we applied the noncentral hypergeometric distribution model implemented in HiEdge (Stav, 2024). Significant interactions were defined using a stringent threshold (q-value <0.001 after multiple testing correction).

This analysis generated paired intrachromosomal interaction networks (one Normal, one TNBC) for each human chromosome. Within these networks, individual nodes represent 40 kb genomic regions, each typically containing one of the following: (a) a transcription start site (TSS) of a protein-coding gene, (2) a non-coding RNA feature (miRNA or lncRNA), or (3) no annotated genomic features (ncDNA) (Supplementary Figure S1C).

### 3.2 TNBC exhibits widespread disruption of intrachromosomal chromatin interactions

Our analysis revealed a genome-wide reduction in chromatin interactions across all chromosomes in TNBC compared to normal tissue. This finding emerged from our systematic comparison of chromosomal interaction patterns, which we quantified both through the absolute number of interactions and their log fold changes between TNBC and normal samples (Figure 1B; Supplementary Figure S1D). We validated that these changes in interaction patterns reflect genuine biological differences rather than technical artifacts: our finding is supported by a Jaccard index analysis of chromosomal node sets (Figure 1B), which demonstrated near-perfect overlap (index ∼1) between TNBC and normal samples, confirming that the same genomic regions are being compared. This widespread loss of interactions showed no correlation with either chromosome size or gene density (Supplementary Figures S2A, B), which suggests a specific biological mechanism. The most pronounced interaction losses were observed in chromosome 18, while chromosome 21 showed the most modest changes, despite their comparable sizes, highlighting the chromosome-specific nature of these alterations.

To further characterize these changes, we performed pairwise Jaccard Similarity analyses of the interaction networks edge sets (Figure 1C). This revealed chromosome-specific patterns of interaction rewiring. We observed that the specific chromatin interactions established in TNBC are changed relative to the Normal interactions as a function of the interaction loss, since the higher Jaccard values match the largest negative log fold change interaction number values. Chromosomes 2, 8, and 4 exhibited unexpectedly low Jaccard indices relative to the other chromosomes with similar levels of interaction loss, which points towards substantial reorganization of their remaining interactions. In contrast, chromosome 19, despite showing considerable interaction loss (logFC <0.50), maintained a relatively conserved pattern of specific interactions.

These findings reveal previously unrecognized complexity in TNBC chromatin architecture, where global interaction loss is followed by chromosome-specific patterns of structural reorganization. While traditional Hi-C contact matrices (Supplementary Figures S3A–C) corroborate this widespread loss of interactions, visual inspection of these heatmaps alone proves insufficient for capturing the full extent of the alterations. The subtle visual differences in these classical representations mask the quantitative changes we detected through or analytical network comparison.

### 3.3 Loss of long-range chromatin interactions in TNBC tissue

**FIGURE 2:**
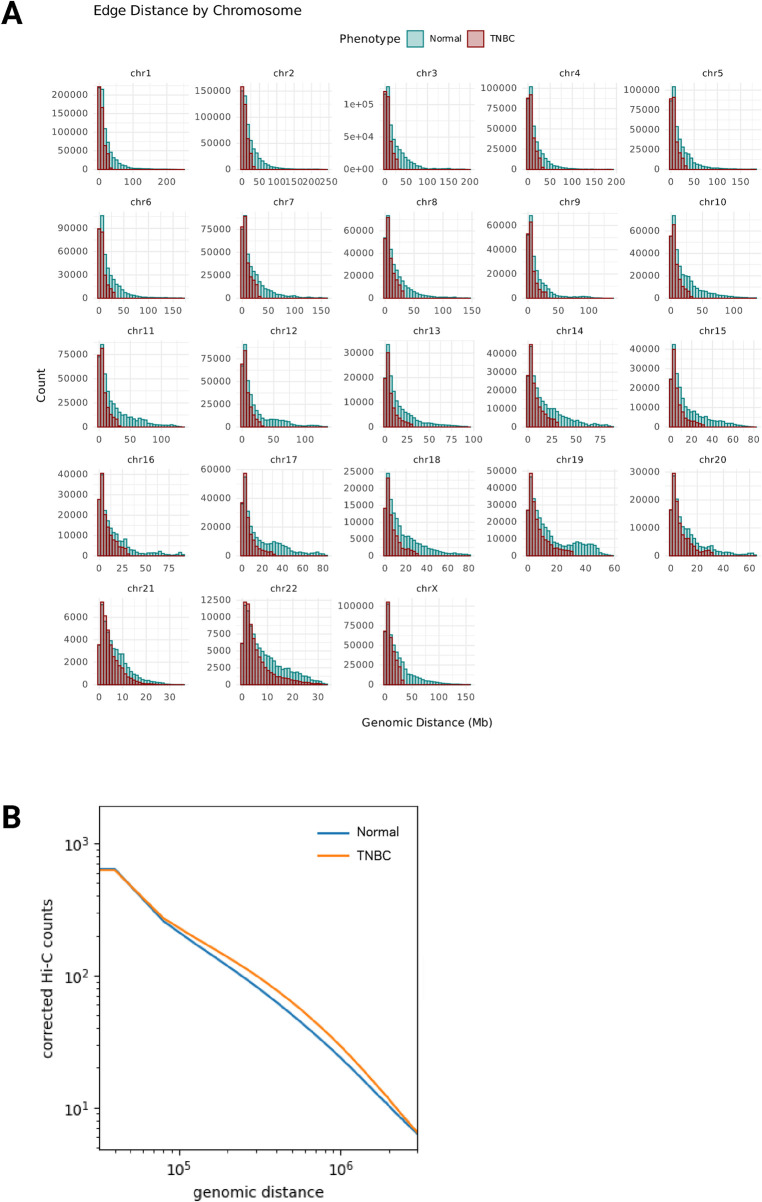
**(A)** Loss of long-range chromatin interactions. Histograms by chromosome of the number of chromatin interactions that occur at each genomic distance (DNA Mb) between nodes in the Normal network (green) and nodes in the TNBC network (dark red). **(B)** Genomic distance (basepairs) versus Hi-C corrected counts plot for all combined intrachromosomal interactions.

Analysis of the edge distance attribute distributions revealed a distinct pattern between Normal and TNBC networks. TNBC showed a marked reduction in significant long-range interactions (>50 Mb), particularly evident in larger chromosomes (Figure 2A). This pattern was consistent across all edge types, whether the nodes they connect contain protein-coding genes, noncoding RNAs, or regions without annotated features (Supplementary Figure S4). This observation is interesting given the biological potential of long-range chromatin contacts, which can mediate enhancer-promoter interactions and in some cases are associated with disease-relevant GWAS variants in non-coding regions, including examples in other breast cancer molecular phenotypes (Dryden et al., 2014; Baxter et al., 2018; Beesley et al., 2020).

Interestingly, at first our findings appear to differ from previous studies surveying Hi-C in other breast cancer models, where increased contact probabilities were reported at the 1–10 Mb range or “long-range interactions”. Kim et al. observed higher relative contact probability at these distances in HCC70 and BT549 TNBC cell lines compared to HMEC normal mammary epithelial cells (Kim et al., 2022). However, these studies analyzed contact probability distributions from corrected Hi-C counts with no confidence or estimate of significance, while our network-based approach focuses on statistically significant interactions (q < 0.001) by modeling the probability of finding the observed number of interactions using the non-central hypergeometric distribution. The methodological and distance range differences are crucial, as classical visualization of the log2 fold-change matrices (TNBC/Normal) from corrected Hi-C counts (Supplementary Figure S3C) confirms the depletion of interactions at extreme distances in TNBC tissue, seen as negative fold-changes (dark blue) in matrix regions far from the diagonal. Indeed, when we examined the relationship between genomic distance and corrected Hi-C counts (Figure 2B), we observe that TNBC tissue shows higher relative contact probability in the 1–10 Mb range compared to the normal tissue, a pattern that was consistent across all chromosomes (Supplementary Figures S3D–F; Supplementary Figure S5).

Previous work in another model comparing normal breast cells (MCF10A) to luminal breast cancer cells (MCF7) reported that long-range interactions at very large genomic distances (>200 Mb) were uniquely present in normal cells. This preservation of long-range contacts in normal cells mirrors our observations in healthy breast tissue samples compared to TNBC tissue samples, suggesting that the loss of these ultra long-range interactions may be a feature of malignant transformation across breast cancer. These findings parallel our lab’s previous observations regarding gene co-expression patterns across multiple cancer types, including breast cancer. We have consistently reported a cancer-specific reorganization pattern characterized by increased local and decreased long-range gene-gene transcriptional relationships (García-Cortés et al., 2020; 2021; 2022; Dorantes-Gilardi et al., 2021). The current Hi-C network analysis provides towards physical evidence supporting these expression-based findings, suggesting a principle of genome reorganization in breast cancer that manifests both functionally and structurally.

### 3.4 Intrachromosomal chromatin interactions are strengthened in TNBC

**FIGURE 3:**
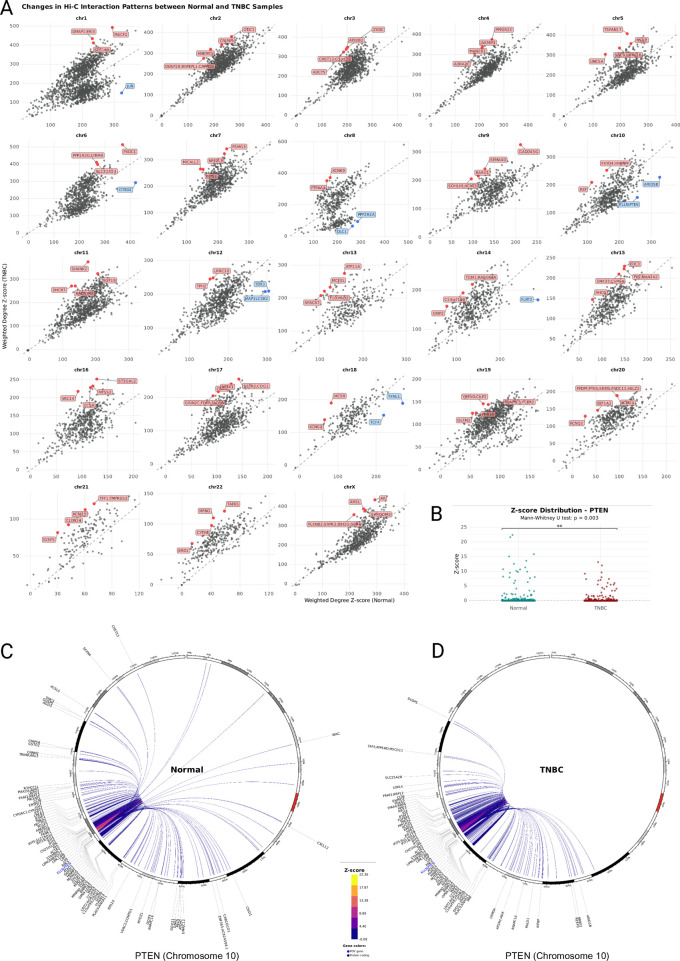
**(A)** Scatterplot of Z-Score weighted node degree. Each panel corresponds to the nodes of the chromosome’s intrachromosomal network. In each plot, one point is a node in the network and its position corresponds to the sum of its edges Hi-C interaction count Z-Score in the normal network (x-axis) and the TNBC network (y-axis). Nodes above the x = y line gain chromatin interaction strength in TNBC and nodes below the x = y line lose chromatin interaction strength in TNBC. The genes within nodes with the largest overall chromatin strength difference in TNBC are indicated in red (gain) or blue (loss) and labeled. **(B)** Z-score distribution dot plot comparing PTEN’s interactions between Normal and TNBC. **(C)** and **(D)** Chromosome 10 chord diagram with the node containing the PTEN gene as the point-of-view (blue). The chromosome’s coordinates increase clockwise, and the first base pair position is at the top. PTEN’s top 200 (Z-score) intrachromosomal interactions with other nodes in normal breast tissue **(C)** and TNBC **(D)** are drawn. Edge color reflects Hi-C interaction Z-Score value. The genes within each node are labeled.

We plotted each node’s Hi-C interaction count z-score weighted degree in the TNBC network compared to the Normal network and found that the nodes in each chromosome’s cancer network follow a different pattern (Figure 3A). Most of the nodes of the chromosomes 2, 3, 7, 9, 14, 15, 16, 19, 20, 21, and 22 Hi-C networks are positioned above the identity line, their intrachromosomal interactions have overall higher normalized Hi-C count values in cancer, despite the general loss of interactions. For chromosomes 1, 4, 5, 6, 8, 10, 11, 12, 13, 17, 18, and X, we observed an additional set of Hi-C nodes below the identity line, indicating a decrease of Hi-C count values in TNBC. We then determined the nodes with the absolute largest distance from the identity line and found that in many cases they contain genes previously reported to play a role specifically in the context of TNBC (Supplementary Table S1), including the tumor suppressor gene PTEN (Chai et al., 2022) in chromosome 10 (Figures 3B–D), DHCR7 in chromosome 11, and the Androgen Receptor (AR) in chromosome X (Supplementary Figure S6). We also found within the most altered nodes, genes that have not been previously reported in the context of TNBC but have been reported in other cancer types, for example, the Microtubule Actin Crosslinking Factor 1 (MACF1) in chromosome 1, and olfactomedin 2 (OLFM2) in chromosome 19. Overall, most of the genome changed its chromatin interaction profile (i.e., in all cases few nodes are on the identity line), and we observed an increased Hi-C interaction count z-score distribution in all the TNBC networks compared to the Normal networks (Supplementary Figure S7). This observation together with the fact that the mean node degree is lower in all the TNBC distributions (Supplementary Figure S8), means that the cancer genome retains strong local chromatin interactions and discards a large proportion of weaker interactions.

### 3.5 The TNBC genome preserves the chromatin interactions of specific genes

**FIGURE 4:**
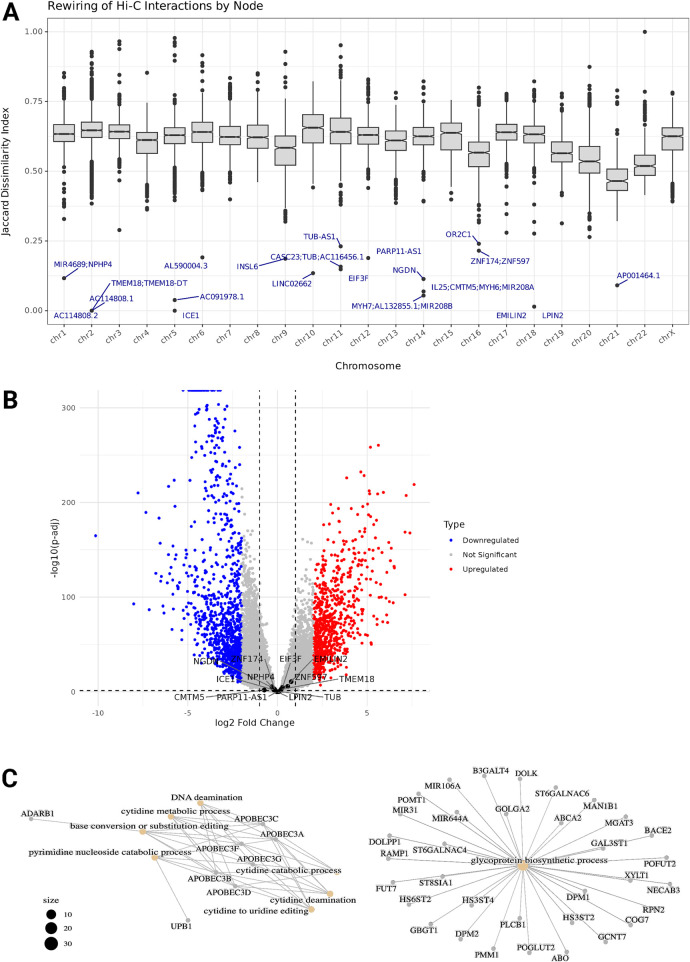
**(A)** Boxplots of Dissimilarity Index distribution from each chromosome's intrachromosomal interactions. The Dissimilarity index ranges from 0 to 1, values closer to 0 indicate a node has preserved its chromatin interactions with the same partner nodes in the TNBC Network compared to the Normal network. The genes within the nodes with the lowest DI are labeled. **(B)** Volcano plot showing differential gene expression between TNBC and Normal samples. Genes with the lowest DI are labeled. **(C)** Functional enrichment of the set of genes preserving their chromatin interactions profile. Network visual representation of the enriched Gene Ontology (GO) terms. Edges connect GO terms that share gene annotations highlighting functional clusters and relationships between the biological processes and molecular functions.

While extensive remodeling of the three-dimensional genome structure was observed across most regions in TNBC, our analysis identified a subset of genomic regions that maintained their chromatin interaction profiles. Using Jaccard dissimilarity index (DI) analysis, we quantified the degree of interaction profile preservation across the genome and identified nodes with the lowest dissimilarity scores, representing the most conserved chromatin architecture (Figure 4A). The dissimilarity index ranged from 0 to 1, with values closer to 0 indicating a node has preserved its exact chromatin interactions in the TNBC Network compared to the Normal network. The DI mean of every chromosome is above 0.5, so for a given node there is a significant rewiring of its intrachromosomal contacts, except for chromosome 21, whose network also has the smaller loss of interactions. Three nodes in chromosome 2, one node in chromosome 5, and one node in chromosome 18 have DI values equal to or very close to zero, meaning their intrachromosomal interactions are intact in the TNBC genome. The genes within these nodes include Transmembrane Protein 18 (TMEM18) and the lncRNA TMEM18 Divergent Transcript (TMEM18-DT), Interactor Of Little Elongation Complex ELL Subunit 1 (ICE1), Elastin Microfibril Interfacer 2 (EMILIN2), and Phosphatidate Phosphatase LPIN2. None of these showed statistically significant differential expression (Figure 4B).

To determine an optimal threshold for selecting highly preserved regions, we implemented an elbow point detection algorithm on the ranked dissimilarity scores. This approach identified a dissimilarity threshold of approximately 0.4, below which genes were considered to have preserved chromatin interactions (Supplementary Figure S9A). We analyzed 1,395 genes throughout all the chromosomes contained within the 1,270 nodes (5.2% of the total nodes) that below the threshold and notably, genes within these conserved regions demonstrated specific functional enrichment patterns.

Gene Ontology (GO) enrichment analysis of the genes located in the most preserved regions revealed significant enrichment of several biological processes (Figure 4C). These processes were primarily related to nucleic acid metabolism and modification, particularly focusing on cytidine processing. The most significantly enriched pathways included cytidine deamination, cytidine to uridine editing, and pyrimidine nucleoside catabolic processes, highlighting the importance of RNA editing mechanisms in these regions with conserved chromatin interactions. Additionally, we observed enrichment in glycoprotein biosynthetic processes and DNA deamination.

Among these genes were several members of the APOBEC family (APOBEC3A, APOBEC3B, APOBEC3C, APOBEC3D, APOBEC3F, and APOBEC3G), which are known to play crucial roles in cytidine deamination and RNA editing. Other notable genes included ADARB1, involved in RNA editing, and various genes associated with glycosylation processes (B3GALT4, POMT1, and DOLK). This functional conservation of chromatin architecture around genes involved in RNA editing and modification suggests that these biological processes may be particularly important for maintaining TNBC cellular identity and function despite broader genomic architectural changes.

### 3.6 Multilayer networks reveal differential reorganization of chromatin structure and gene expression in TNBC

We constructed multilayer networks integrating Hi-C and gene co-expression data for both normal TNBC. These networks consisted of two interconnected layers: the Hi-C layer representing chromatin interactions between the 40 kb genomic regions and the Mutual Information layer representing gene co-expression relationships. The 40 kb resolution was selected as it represents an optimal balance where most bins contain a single gene’s transcription start site (Supplementary Figure S9B), enabling a mapping between chromatin structure and gene regulation. Genes in the MI layer were connected to their corresponding genomic regions in the Hi-C layer through interlayer edges.

**FIGURE 5:**
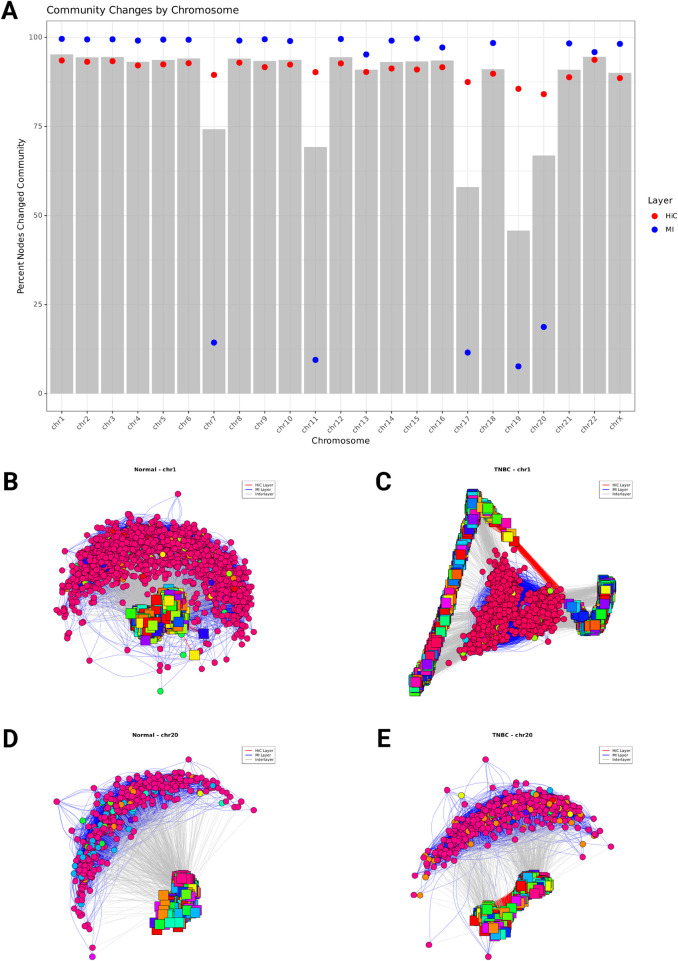
**(A)** Percentage of Hi-C Network nodes changing community membership between Normal and TNBC across all chromosomes. The bar plots represent the overall percentage of nodes that changed community assignment. Red points indicate the percentage of Hi-C nodes that changed community membership, and blue points show the percentage of MI nodes (gene coexpression) that changed. **(B–D)** Multilayer network community structure for chromosome 1 **(B, C)** and chromosome 20 **(D, E)**. Nodes represent either chromatin interaction 40 kb genomic regions (squares) or genes (circles), with edges indicating interactions between nodes. Red edges connect chromatin regions (Hi-C layer), blue edges connect co-expressed genes (MI layer), and gray edges represent interlayer connections between genes and their corresponding genomic regions. Node colors indicate community membership, with each community shown in a distinct color. The network layout is generated using the Fruchterman-Reingold algorithm.

Our community detection analysis revealed substantial reorganization of network structure between normal and TNBC conditions across all chromosomes (Figure 5A). On average, 86% of nodes changed their community membership between conditions, indicating widespread rewiring of functional genomic modules during cancer progression. When analyzing layer-specific changes, we found that the Hi-C layer consistently showed a higher percentage of nodes changing community membership (90.8% on average, red points) compared to the MI layer (79.8% on average, blue points), suggesting that chromatin structure undergoes more extensive reorganization than gene expression patterns in TNBC.

We observed chromosome-specific patterns in the extent of layer-specific reorganization. While some chromosomes exhibited concurrent changes in both chromatin structure and gene expression, others showed divergent patterns. For example, chromosome 1 (Figures 5B,C) displayed substantial community restructuring in both layers, with 93% of Hi-C nodes and 99% of MI nodes changing their community membership. The multilayer network of chromosome 1 reveals 13 major communities in normal tissue that reorganize into 17 communities in TNBC, with significant rewiring of connections both within and between layers. In contrast, chromosome 20 (Figures 5D,E) exhibited a disparity between layers, with 84% of Hi-C nodes changing community membership while only 18.7% of MI nodes were affected. This suggests that at the chromosome level extensive reorganization of chromatin structure in chromosome 20 occurs without corresponding changes in gene co-expression patterns. Chromosome 20s network structure maintains 11 distinct communities in both normal tissue and TNBC, though the composition of these communities undergoes significant restructuring occurs within the Hi-C layer, while the MI layer exhibits relatively stable community organization despite the disease state transition.

These chromosome-specific patterns of community reorganization suggest that the relationship between chromatin structure alterations and gene expression changes is not uniform across the genome. Some chromosomal regions exhibit coordinated changes across both regulatory layers, while others show more independent reorganization patterns. This heterogeneity in multilayer network restructuring may reflect different mechanisms of gene regulation disruption in cancer, with some regions experiencing primarily structural reorganization and others undergoing more complex rewiring involving both chromatin structure and transcriptional programs.

### 3.7 Global structure-expression correlation patterns

**FIGURE 6:**
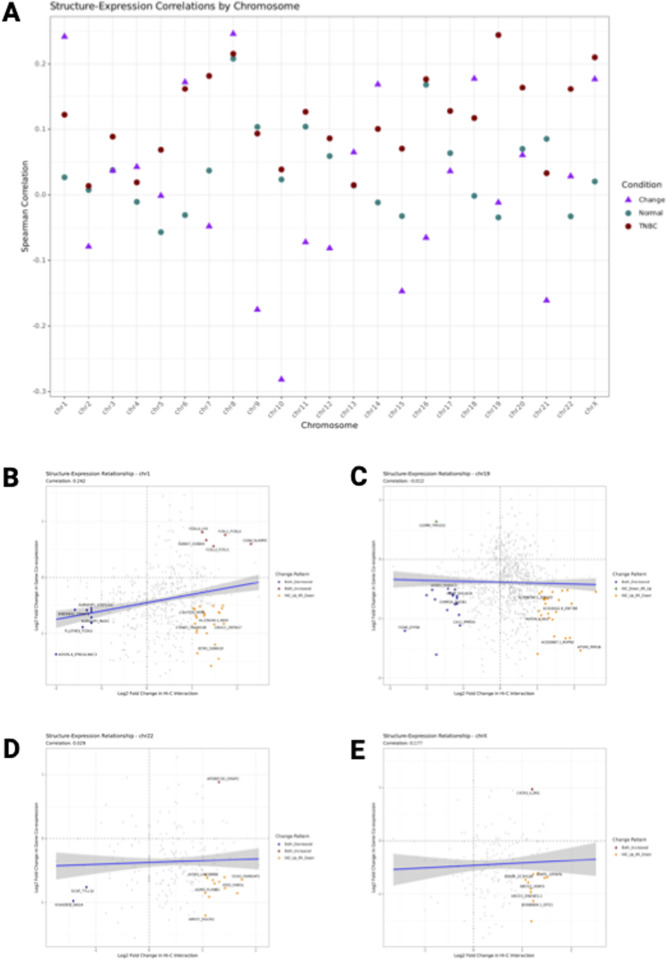
Structure-Expression correlation. **(A)** Correlation coefficients between chromatin interaction strength and gene co-expression across all chromosomes. Green points represent correlation coefficients in Normal breast tissue, red points show correlations in TNBC, and purple triangles indicate correlations between changes in Hi-C and MI values between conditions. **(B–E)** Relationship between chromatin structure and gene expression changes in Normal versus TNBC for chromosomes 1 **(B)**, 19 **(C)**, 22 **(D)**, and X **(D)**. Each point represents a gene pair, with the x-axis showing log2 fold change in chromatin interaction strength (Hi-C layer) and the y-axis showing log2fold change in gene co-expression (Mi layer) between Normal and TNBC. Gray points indicate all gene pairs, colored points highlight significant changes (red: both Hi-C and MI increased; blue: both decreased; orange: Hi-C increased and MI decreased; green: Hi-C decreased and MI increased). The blue line represents the linear regression fit with 95% confidence interval. The correlation coefficient (r) is show in the subtitle. Dashed lines divide the plot into quadrants representing different patterns of change.

Our analysis of the relationship between chromatin interaction strength and gene co-expression revealed distinct correlation patterns across chromosomes in both normal breast tissue and TNBC (Figure 6A). In normal tissue (green points), we observed positive correlations between chromatin interaction strength and gene co-expression for most chromosomes, with correlation coefficients ranging from 0.18 to 0.42. In TNBC (red points), these correlations were generally maintained but with some chromosome-specific variations, ranging from 0.15 to 0.45.

Interestingly, the correlation between changes in Hi-C and changes in MI values between conditions (purple triangles) showed a different pattern. These change correlations, which capture how alterations in chromatin structure relate to changes in gene expression during the transition from normal to TNBC, ranged from 0.21 to 0.39 across chromosomes. This suggests that while chromatin structure and gene expression are generally correlated within each condition, the relationship between their changes during cancer progression is more complex and varies by chromosome.

### 3.8 Chromosome-specific structure-expression relationships

Detailed analysis of individual chromosomes revealed striking differences in structure-expression relationships (Figures 6B–E). Chromosome 1 (Figure 6B) showed a moderate correlation (r = 0.28) between changes in chromatin interaction strength and gene co-expression, with a relatively balanced distribution of gene pairs across the four possible patterns of change. In contrast, chromosome 19 (Figure 6C) exhibited a stronger correlation (r = 0.36), with a notable enrichment of gene pairs showing concordant changes (both increased or both decreased). Chromosome 22 (Figure 6D) displayed one of the strongest correlations (r = 0.39), with a particularly high proportion of gene pairs showing concordant decreases in both chromatin interaction and gene co-expression. The X chromosome (Figure 6E) presented a unique pattern with a lower correlation coefficient (r = 0.23) and a more dispersed distribution of gene pairs, suggesting a less coordinated relationship between chromatin structural changes and gene expression alterations in this sex chromosome.

### 3.9 Gene pair-specific effects diverge from global patterns

While the global analysis showed positive correlations between chromatin structure and gene expression changes, examination of individual gene pairs revealed more nuanced patterns that often diverged from these global trends. Across all chromosomes, we identified substantial numbers of gene pairs exhibiting discordant changes (orange and green points in Figures 6B–E), where chromatin interaction strength increased while gene co-expression decreased or *vice versa*.

Specifically, chromosome 1 showed a relatively high proportion of gene pairs with discordant changes, including gene pairs with increased chromatin interaction but decreased gene co-expression (orange points) and gene pairs with decreased chromatin interaction but increased gene co-expression (green points). This suggests that for a substantial subset of gene pairs, the relationship between chromatin structure and gene expression in TNBC is inverse rather than direct. The proportions of gene pairs showing different patterns varied considerably across chromosomes. On chromosome 19, most significantly changed gene pairs showed concordant change, while fewer pairs showed discordant patterns. In contrast, chromosome X had nearly equal proportions of concordant and discordant change patterns, suggesting distinct regulatory mechanisms.

These gene pair-specific effects highlight the complexity of genomic regulation in cancer and demonstrate that while global patterns suggest a positive correlation between chromatin structure and gene expression changes, the relationship at the level of individual gene pairs is more heterogeneous. This divergence between global patterns and gene pair-specific effects underscores the importance of analyzing structure-expression relationships at multiple levels to understand the complex regulatory changes occurring during cancer progression. Overall, our results suggest that TNBC involves complex rewiring of the relationship between chromatin structure and gene expression, with effects that can be concordant or discordant depending on the specific genomic context and gene pairs involved.

## 4 Discussion

In this study, we constructed integrated network graphs from intrachromosomal Hi-C data, that enabled an unbiased, comprehensive examination of chromatin interactions that traditional approaches would miss. We chose to analyze primary tumor samples rather than cell lines, capturing complexity of chromatin organization in actual disease states. Chromatin architecture has previously been shown to influence tumorigenic transcriptional programs. We chose to study TNBC as this type of breast cancer is a major health problem with limited treatment options. Our network-based analysis approach yielded results that align with findings from the original study where we obtained the data (Kim et al., 2022). For instance, despite our focus on integrated network graphs rather than directly examining TADs (Topologically Associated Domains), we similarly observed a decrease in chromatin interactions in TNBC samples. This consistency validates our methodological approach while offering complementary insights through network analysis. The reduction in interactions we detected supports previous observations about altered chromatin architecture in TNBC, contributing to the dysregulated transcriptional programs characteristic of this aggressive cancer subtype.

Our study reveals significant alterations in PTEN chromatin interactions within TNBC tumors, providing a structural basis for the dysregulated PI3K/AKT signaling commonly observed in this aggressive breast cancer subtype. Our observations of altered PTEN chromatin interactions support the mechanistic context of a recently described therapeutic strategy (Schade et al., 2024), as EZH2 inhibition may restore normal chromatin architecture around the PTEN locus, thereby reestablishing tumor suppressor function and enhancing sensitivity to AKT inhibition. This chromatin-level understanding of PTEN regulation offers new insights into how the PI3K pathway might be more effectively targeted in TNBC, particularly in tumors where PTEN function is compromised through epigenetic rather than genetic mechanisms.

We identified patterns of alterations in chromatin structure that directly correlated with changes in gene co-expression networks. Our findings showed an altered chromatin architecture in genes previously implicated in TNBC pathogenesis, providing a mechanistic link between structural changes and phenotypic outcomes. The network approach revealed new structure-function relationships which would not have been discovered by analyzing Hi-C and RNA-seq data separately. This gene-centered approach of mapping genomic features to network nodes revealed biologically relevant insights into the pathophysiology of TNBC. By analyzing Hi-C networks alongside information on regulatory networks inferred from RNA-seq data we created a unified perspective on expression patterns and chromatin structure. This integration showed that certain TNBC-associated genes exhibited coordinated changes in both their spatial organization and transcriptional relationships, suggesting that chromatin restructuring may play a general role in the altered gene expression of TNBC.

The methodological framework we developed in this study offers several technical advantages. First, our approach provides a unified platform that contextualizes genome architecture in relation to transcriptional regulation. Second, it enables both high-resolution gene-level investigation and broader genome-wide analysis within the same analytical framework. Third, by representing complex genomic interactions as network properties, we transform multidimensional Hi-C data into interpretable, quantifiable metrics that facilitate comparison across conditions.

A key advantage of representing Hi-C data as networks is the flexibility in how edge weights can be defined and modified based on specific analytical objectives. While we primarily utilized unweighted networks to capture the fundamental topology of chromatin interactions, our framework allows for dynamic weight assignment using various metrics -from raw interaction frequencies to normalized scores from diverse normalization methods-enabling multi-faceted investigation of chromatin organization. Network representation additionally accommodates variation in Hi-C data resolution, as nodes can be defined at different genomic scales, which would imply a different interpretation of differential and multilayer analyses. For instance, increasing a bin size to 100 kb might reveal higher-order organizational changes while potentially obscuring gene-specific effects, whereas reducing resolution to 10 kb could expose fine-grained regulatory interactions between DNA sequences but might fragment the communities identified at 40 kb resolution that correspond to coordinated gene regulation domains.

Our multilayer network approach offers significant advantages over traditional single-layer analyses by revealing layer-specific changes, as the differential reorganization of Hi-C and MI layers would not be apparent from analyzing either data type in isolation. It also provides a systems-level view: the simultaneous visualization of chromatin interactions, gene co-expression, and their interconnections offers a comprehensive perspective on the genomic regulatory landscape. Importantly, our computational biology approach has generated several observations worthy of experimental follow-up. The patterns of change observed between chromatin interactions (Hi-C) and gene co-expression (MI) networks provide intriguing biological hypotheses. Gene pairs exhibiting increased Hi-C interactions but decreased mutual information (“Hi-C up, MI down”) might represent cases where enhanced chromatin proximity in cancer paradoxically disrupts transcriptional coordination, possibly by preventing access to transcription factors or other DNA-binding proteins. Conversely, gene pairs showing decreased chromatin interactions but increased co-expression (“Hi-C down, MI up”) could indicate regions where chromatin becomes more accessible in cancer, enabling co-regulation of genes that would normally be transcriptionally independent. We also observed cases where both chromatin interactions and transcriptional coordination intensify simultaneously. For example, APOBEC3G (which maintained a largely stable interaction profile) and GRAP2 (GRB2-related adaptor protein) demonstrated concurrent increases in Hi-C interaction strength and mutual information, suggesting an intensification of transcriptional regulation via existing chromatin interaction frameworks. This pattern reveals how cancer can also leverage the existing three-dimensional genome architecture to enhance specific transcriptional programs rather than necessarily remodeling the architecture itself.

While our current study focused on intrachromosomal interactions and therefore on chromosome territories, the network analysis framework we developed could be readily extended to incorporate interchromosomal interactions. Such an extension would provide a more complete picture of the nuclear architecture and potentially uncover long-range regulatory mechanisms that cross chromosomal boundaries. Interchromosomal interactions, though less frequent than intrachromosomal contacts, may play roles in coordinating the expression of functionally related genes located on different chromosomes. A network-based approach is particularly well-suited to capture these complex, multi-chromosomal relationships and could reveal higher-order principles of genome organization relevant to TNBC biology.

Despite these advantages, we acknowledge certain limitations. The resolution of Hi-C data can restrict granularity of the interactions we can detect, although other groups (Pancaldi et al., 2016b) have used targeted chromosome conformation capture techniques like promoter capture to overcome this limitation. Additionally, while network inference algorithms provide valuable insights, they require validation to ensure biological relevance. Future iterations could benefit from incorporating additional epigenomic features as network attributes and developing more sophisticated network topology analyses. Integration of single-cell Hi-C data would also address heterogeneity concerns inherent in bulk tissue analyses. This integrated network approach could be extended to other cancer types, facilitating comparative studies of chromatin reorganization across malignancies. Furthermore, our methodology can leverage growing availability of multi-omics datasets in public repositories, enabling integrative analyses across chromatin conformation, transcriptomics, and epigenomics in various conditions.

## Data Availability

Publicly available datasets were analyzed in this study. The datasets generated and analyzed for this study can be found in the GitHub repositories GSE167150_HiC_matrices (https://github.com/CSB-IG/GSE167150_HiC_matrices) and GSE167150_HiC_networks (https://github.com/CSB-IG/GSE167150_HiC_networks). These repositories contain the code to generate the datasets from the raw data.
