# Supplementary material for: Integration of chromosome conformation and gene expression networks reveals regulatory mechanisms in triple negative breast cancer
Source: Front Cell Dev Biol. 2025 Jul 4;13:1597245. doi: 10.3389/fcell.2025.1597245 (PMC12271745; doi:10.3389/fcell.2025.1597245)

SUPPLEMENTARY FIGURE 1

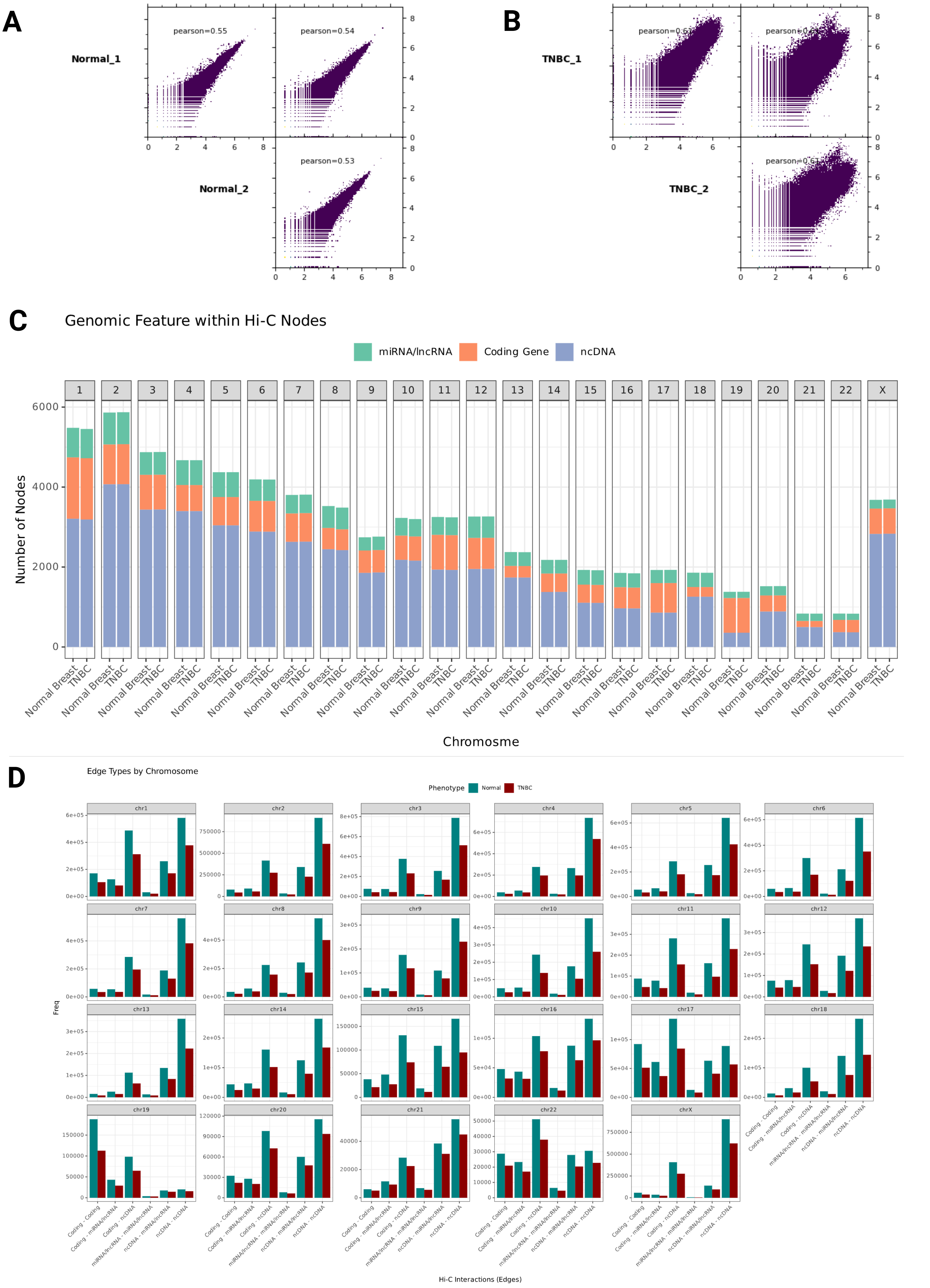

SUPPLEMENTARY FIGURE 2

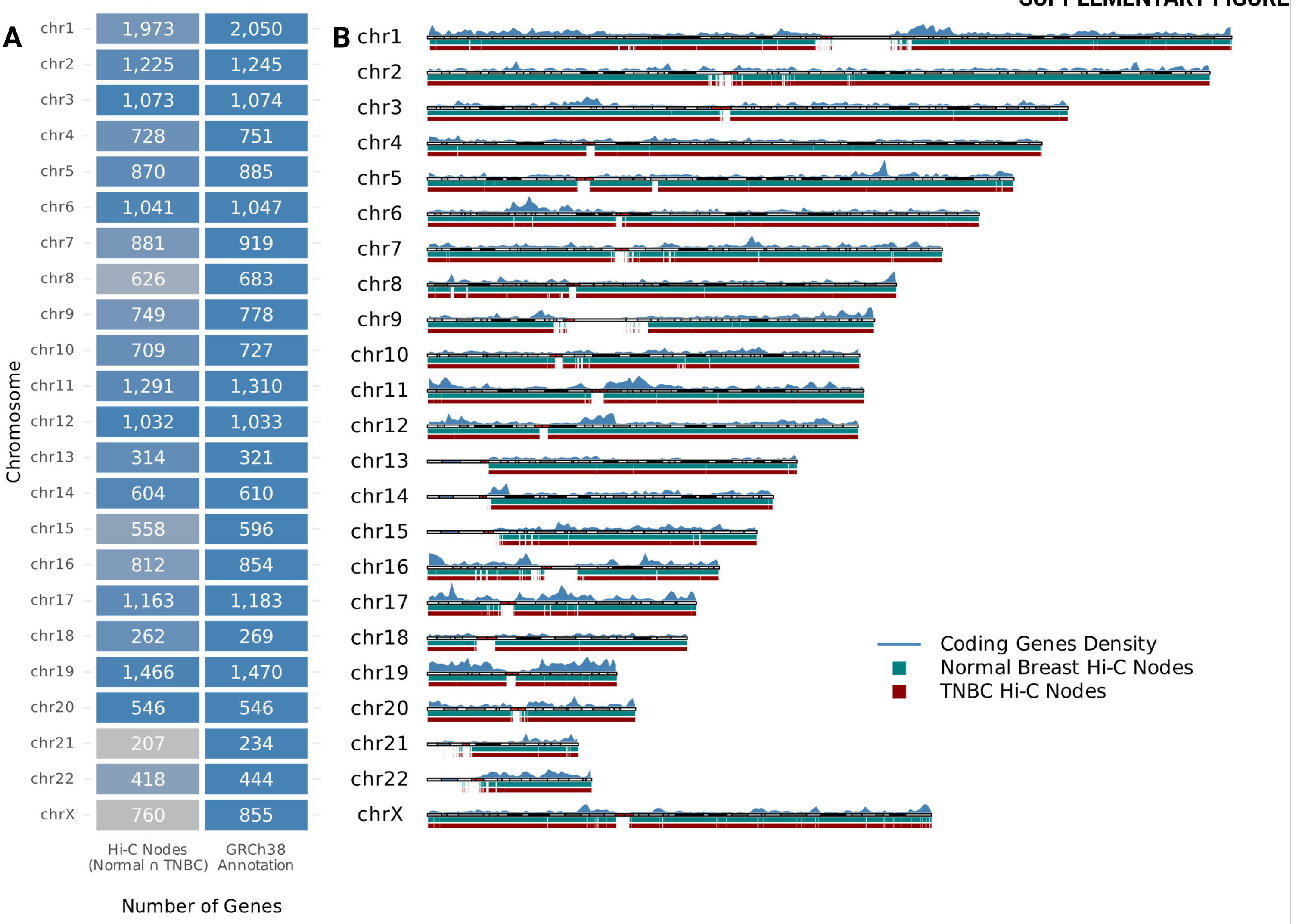

SUPPLEMENTARY FIGURE 3

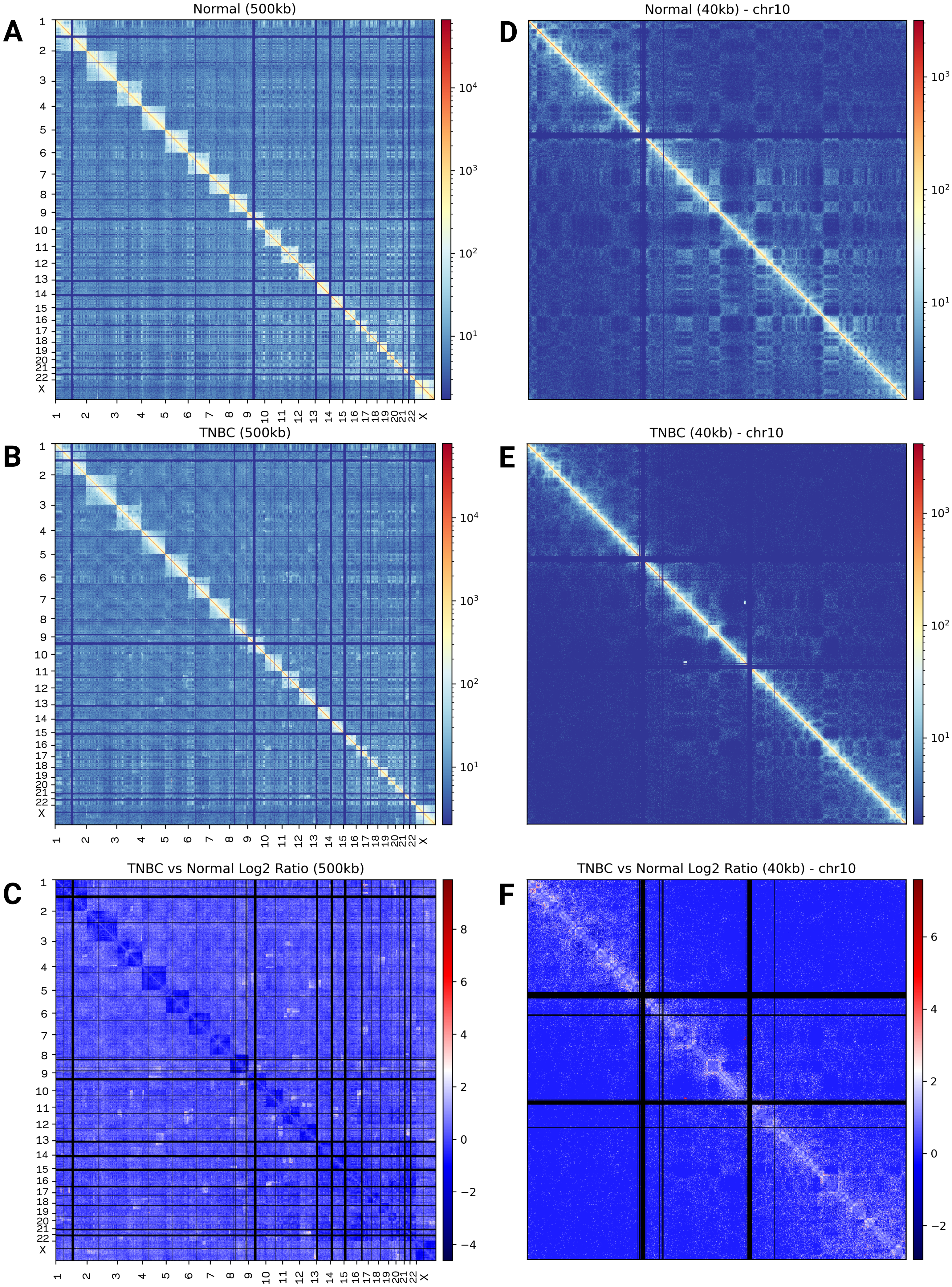

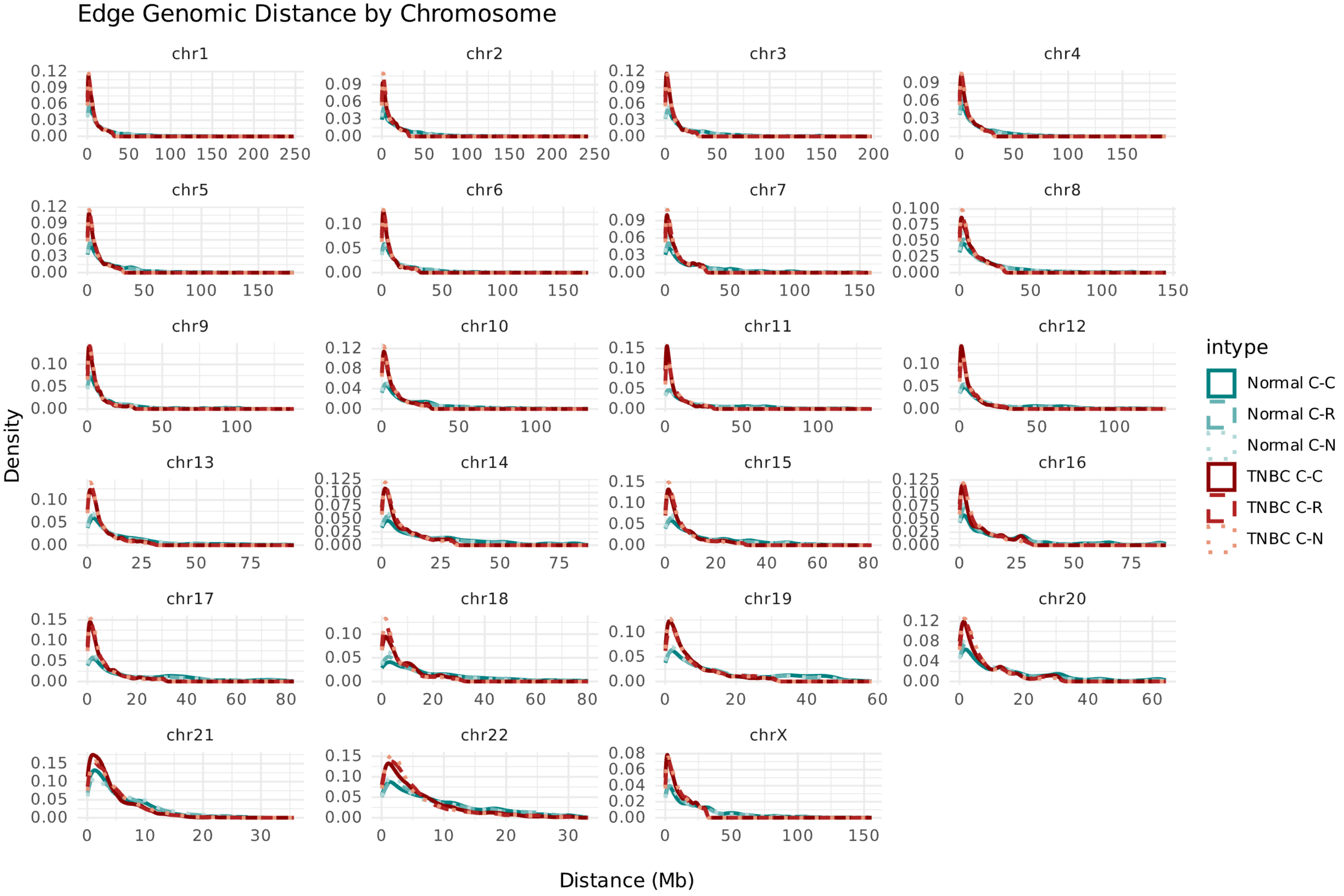
Edge Genomic Distance by Chromosome
chr1
chr2
chr3
chr4
chr5
chr6
chr7
chr8
chr9
chr10
chr11
chr12
chr13
chr14
chr15
chr16
chr17
chr18
chr19
chr20
chr21
chr22
chrX
Density
Distance (Mb)
intype
Normal C-C
Normal C-R
Normal C-N
TNBC C-C
TNBC C-R
TNBC C-N

SUPPLEMENTARY FIGURE 5

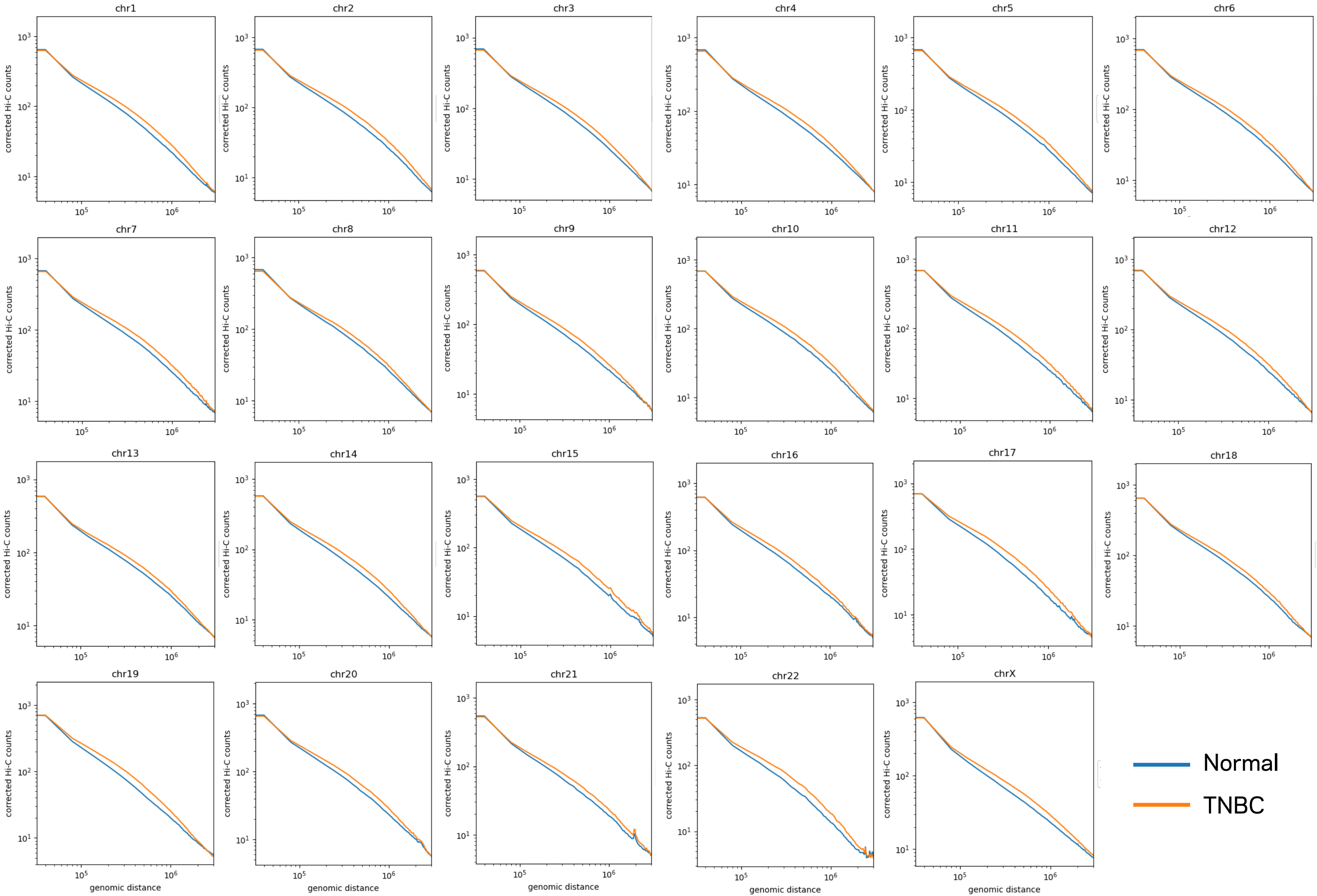

SUPPLEMENTARY FIGURE 6

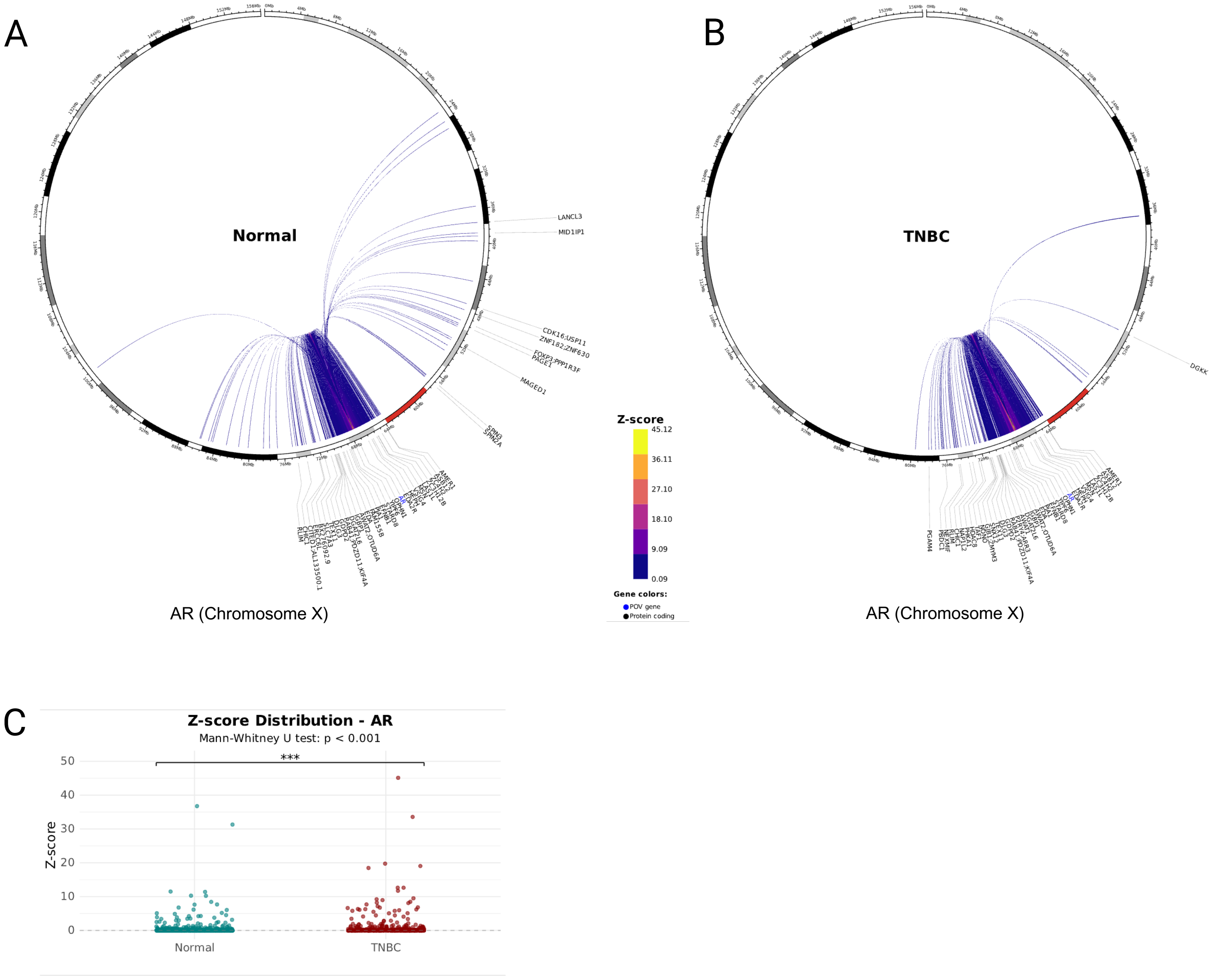

SUPPLEMENTARY FIGURE 7

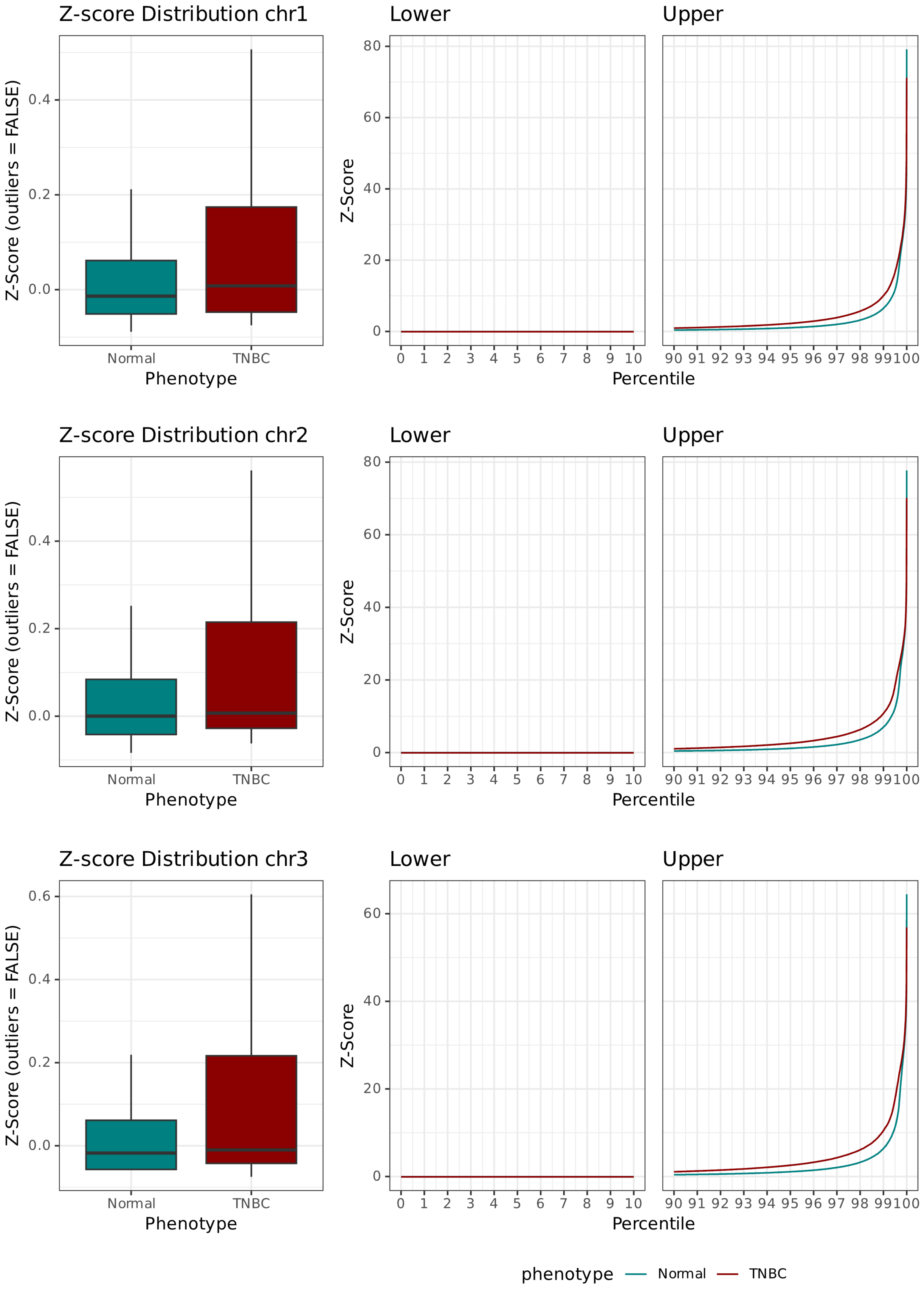

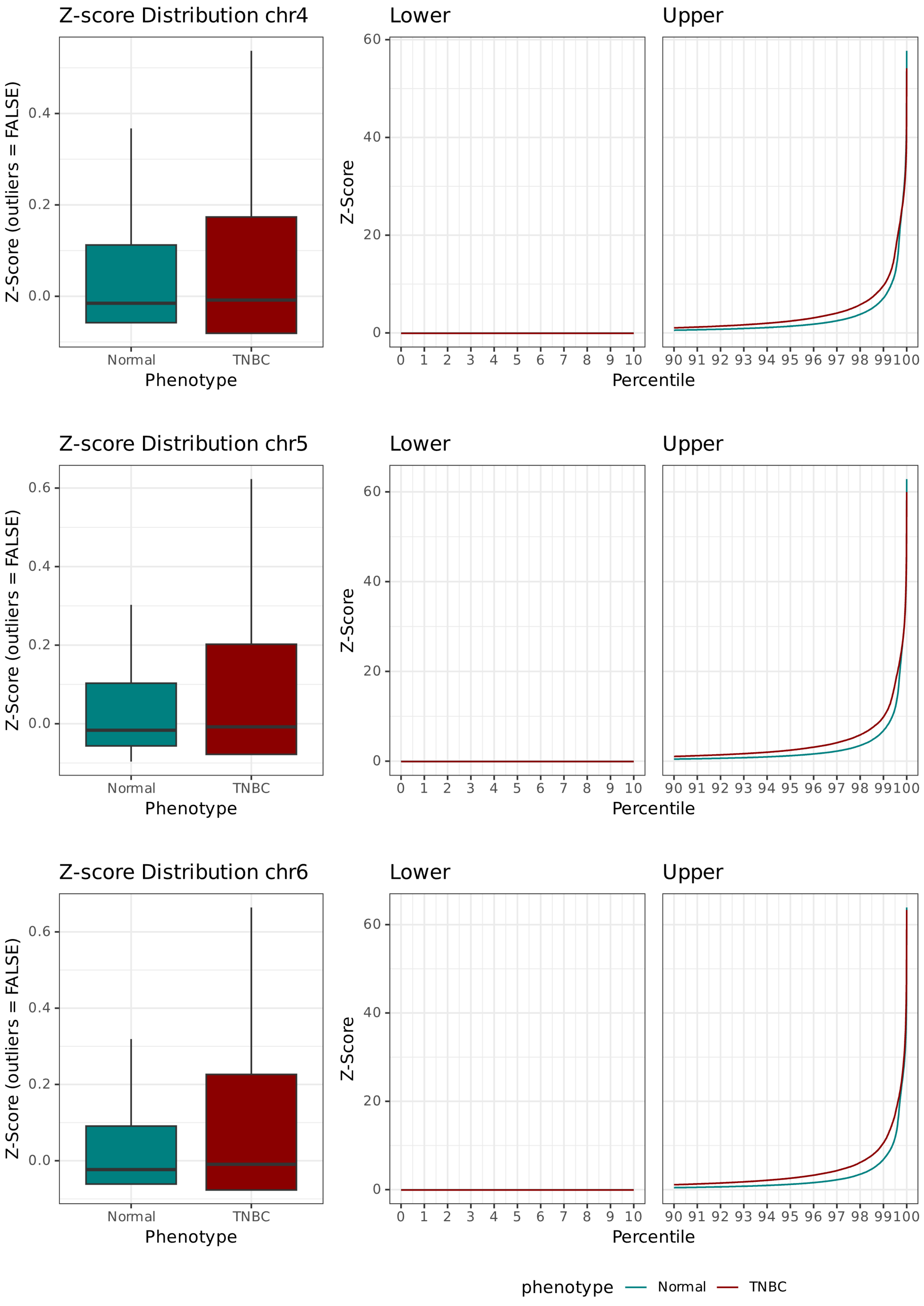
Z-score Distribution chr4
Lower
Upper
Z-Score (outliers = FALSE)
0.0
0.2
0.4
Normal
TNBC
Phenotype
Z-Score
0
20
40
60
0 1 2 3 4 5 6 7 8 9 10
90 91 92 93 94 95 96 97 98 99100
Percentile
Z-score Distribution chr5
Lower
Upper
Z-Score (outliers = FALSE)
0.0
0.2
0.4
0.6
Normal
TNBC
Phenotype
Z-Score
0
20
40
60
0 1 2 3 4 5 6 7 8 9 10
90 91 92 93 94 95 96 97 98 99100
Percentile
Z-score Distribution chr6
Lower
Upper
Z-Score (outliers = FALSE)
0.0
0.2
0.4
0.6
Normal
TNBC
Phenotype
Z-Score
0
20
40
60
0 1 2 3 4 5 6 7 8 9 10
90 91 92 93 94 95 96 97 98 99100
Percentile
phenotype
Normal
TNBC

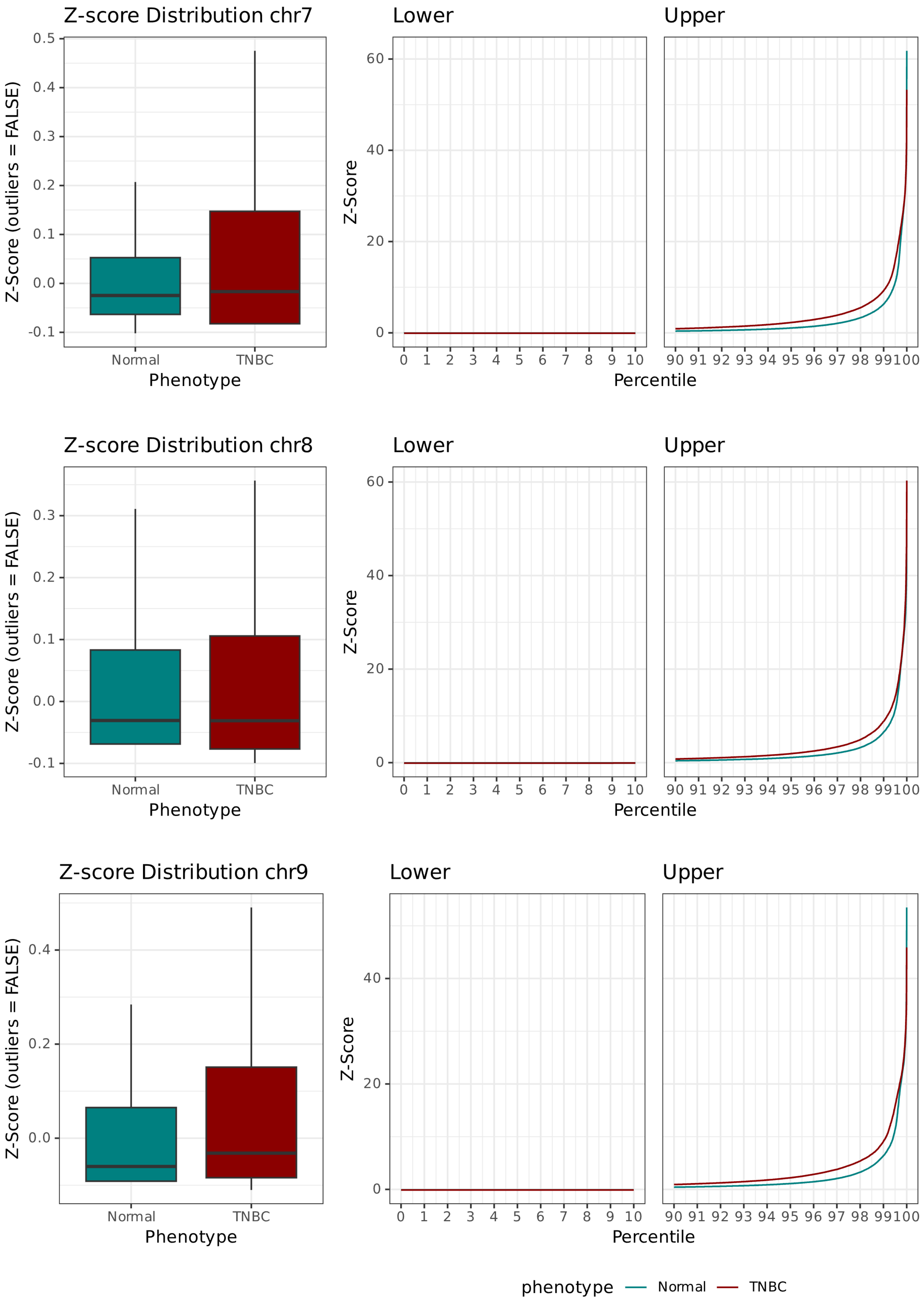
Z-score Distribution chr7
Z-Score (outliers = FALSE)
0.5
0.4
0.3
0.2
0.1
0.0
-0.1
Normal
TNBC
Phenotype
Lower
Upper
Z-Score
60
40
20
0
0 1 2 3 4 5 6 7 8 9 10
90 91 92 93 94 95 96 97 98 99 100
Percentile
Z-score Distribution chr8
Z-Score (outliers = FALSE)
0.3
0.2
0.1
0.0
-0.1
Normal
TNBC
Phenotype
Lower
Upper
Z-Score
60
40
20
0
0 1 2 3 4 5 6 7 8 9 10
90 91 92 93 94 95 96 97 98 99 100
Percentile
Z-score Distribution chr9
Z-Score (outliers = FALSE)
0.4
0.2
0.0
Normal
TNBC
Phenotype
Lower
Upper
Z-Score
40
20
0
0 1 2 3 4 5 6 7 8 9 10
90 91 92 93 94 95 96 97 98 99 100
Percentile
phenotype
Normal
TNBC

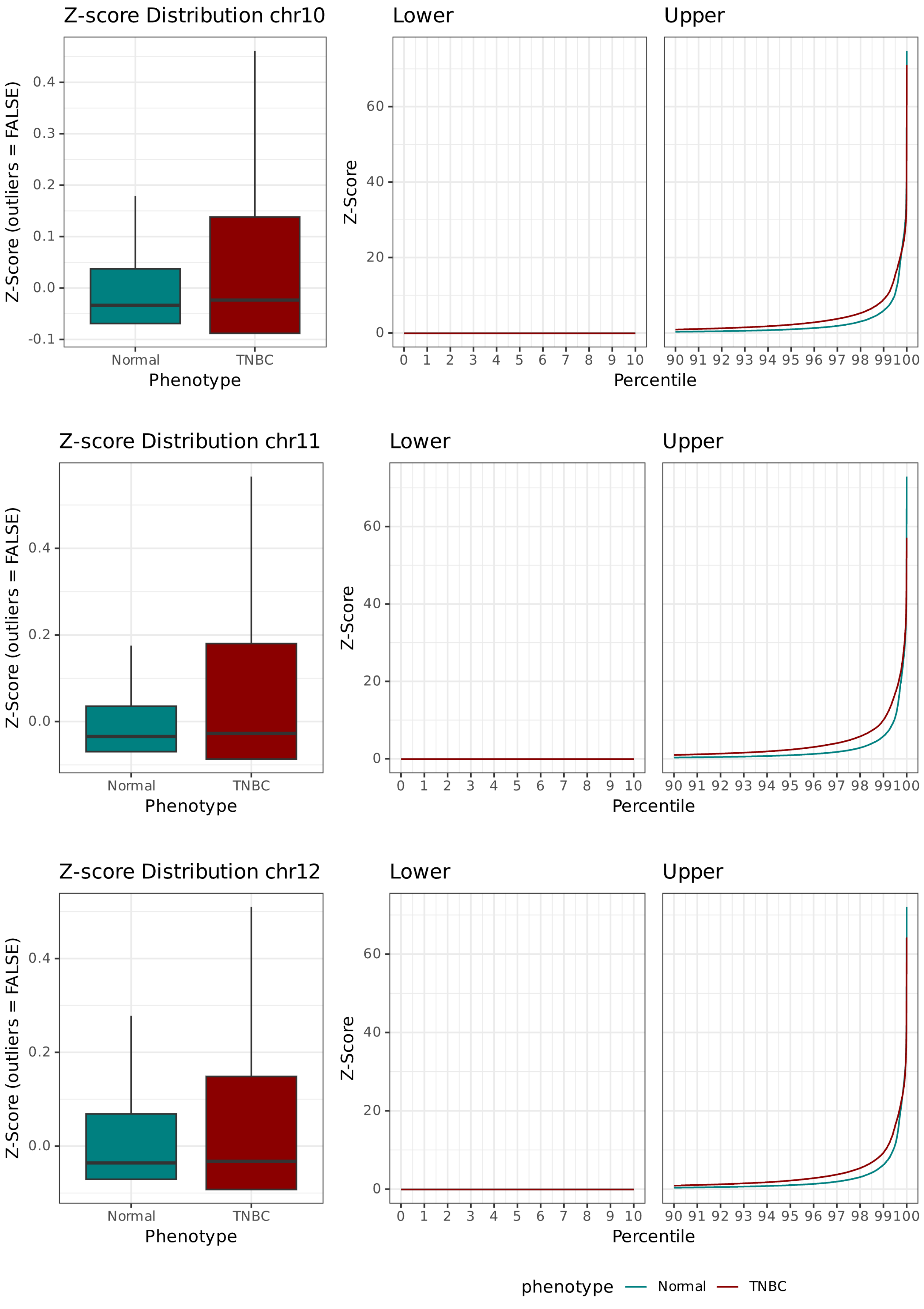
Z-score Distribution chr10
Z-Score (outliers = FALSE)
0.4
0.3
0.2
0.1
0.0
-0.1
Normal
TNBC
Phenotype
Lower
Upper
Z-Score
60
40
20
0
0 1 2 3 4 5 6 7 8 9 10
90 91 92 93 94 95 96 97 98 99 100
Percentile
Z-score Distribution chr11
Z-Score (outliers = FALSE)
0.4
0.2
0.0
Normal
TNBC
Phenotype
Lower
Upper
Z-Score
60
40
20
0
0 1 2 3 4 5 6 7 8 9 10
90 91 92 93 94 95 96 97 98 99 100
Percentile
Z-score Distribution chr12
Z-Score (outliers = FALSE)
0.4
0.2
0.0
Normal
TNBC
Phenotype
Lower
Upper
Z-Score
60
40
20
0
0 1 2 3 4 5 6 7 8 9 10
90 91 92 93 94 95 96 97 98 99 100
Percentile
phenotype
Normal
TNBC

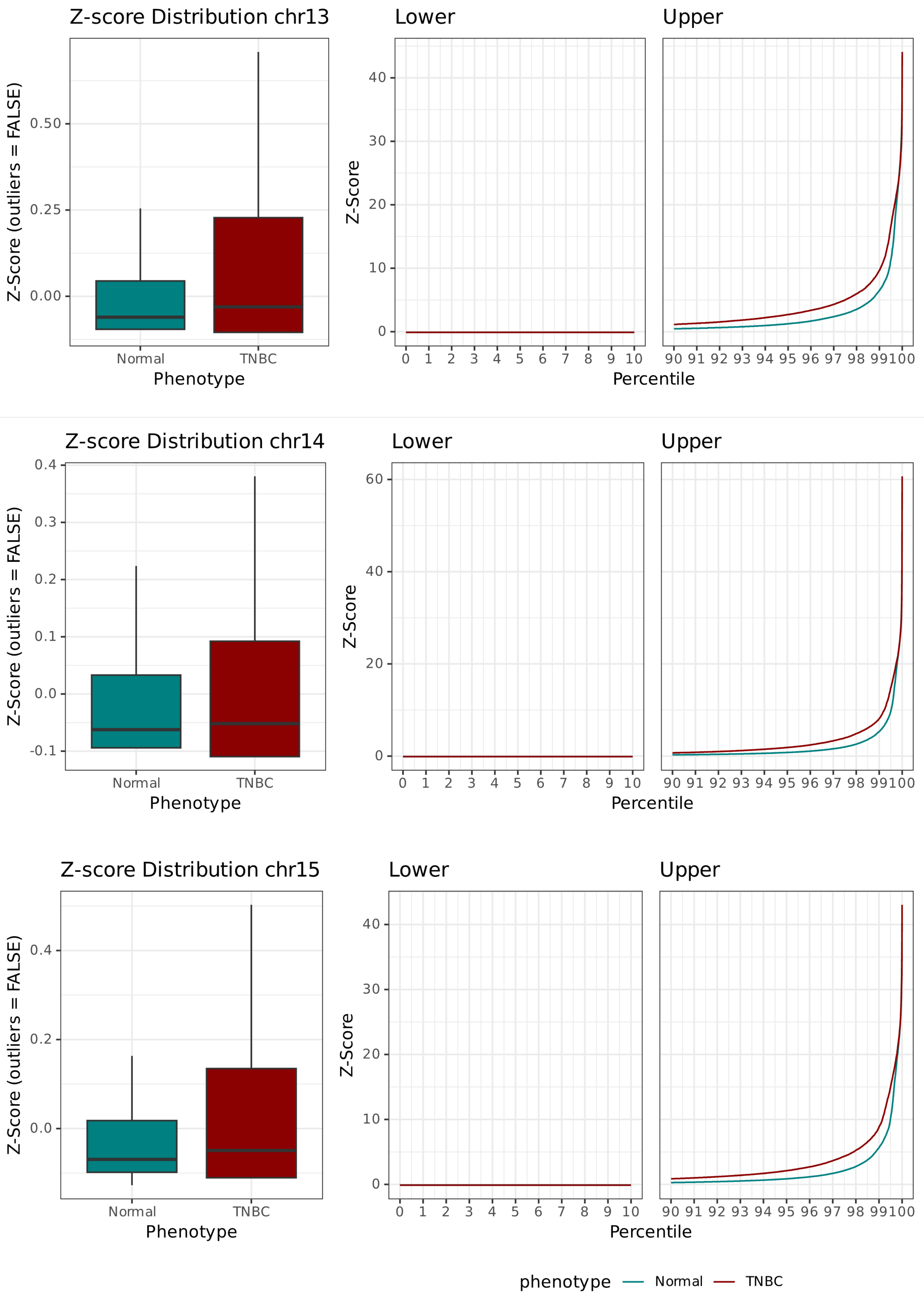
Z-score Distribution chr13
Lower
Upper
Z-Score (outliers = FALSE)
0.00
0.25
0.50
Normal
TNBC
Phenotype
Z-Score
0
10
20
30
40
0 1 2 3 4 5 6 7 8 9 10
90 91 92 93 94 95 96 97 98 99 100
Percentile
Z-score Distribution chr14
Lower
Upper
Z-Score (outliers = FALSE)
-0.1
0.0
0.1
0.2
0.3
0.4
Normal
TNBC
Phenotype
Z-Score
0
20
40
60
0 1 2 3 4 5 6 7 8 9 10
90 91 92 93 94 95 96 97 98 99 100
Percentile
Z-score Distribution chr15
Lower
Upper
Z-Score (outliers = FALSE)
0.0
0.2
0.4
Normal
TNBC
Phenotype
Z-Score
0
10
20
30
40
0 1 2 3 4 5 6 7 8 9 10
90 91 92 93 94 95 96 97 98 99 100
Percentile
phenotype
Normal
TNBC

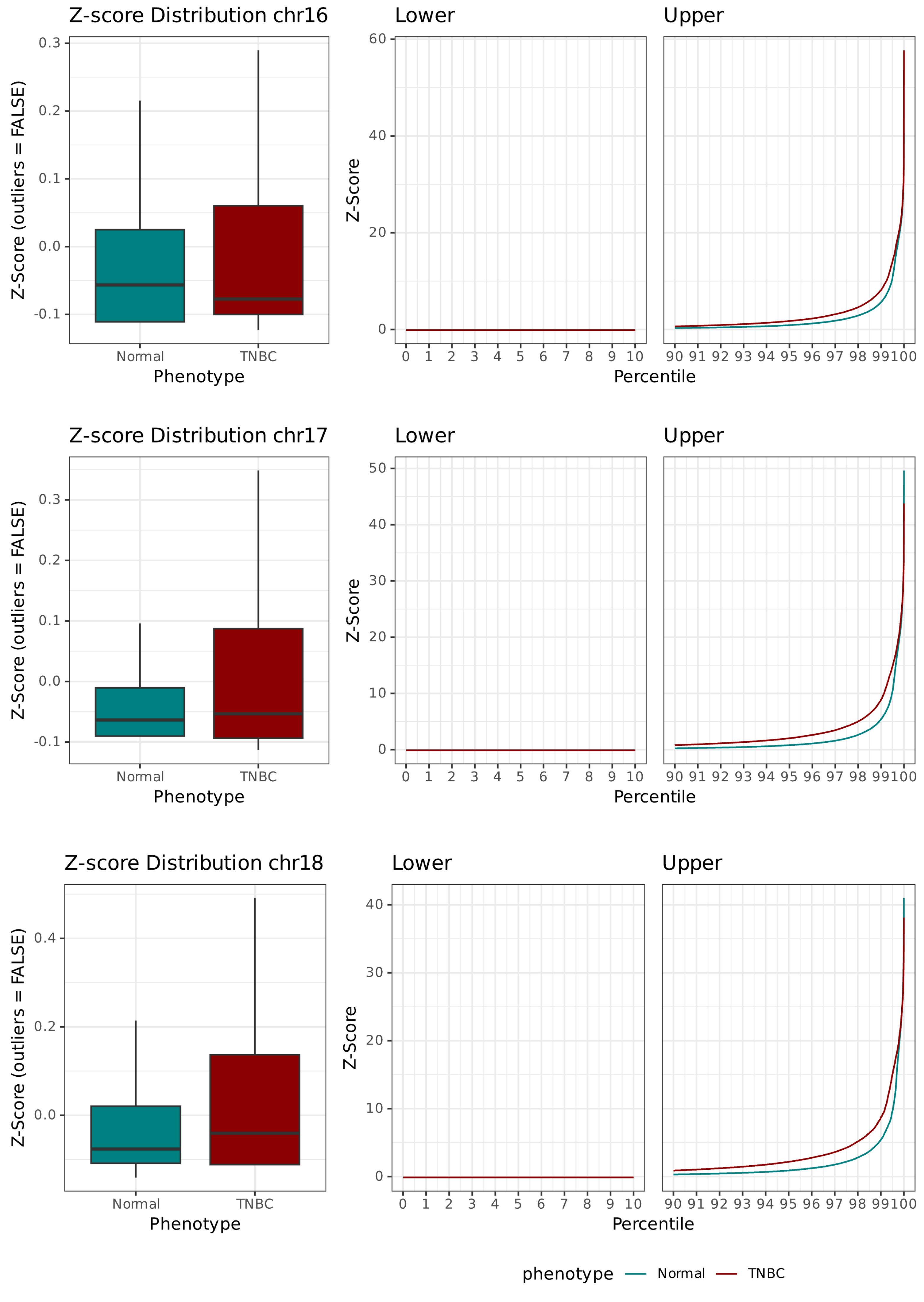

Z-score Distribution chr16
Z-Score (outliers = FALSE)
0.3
0.2
0.1
0.0
-0.1
Normal
TNBC
Phenotype
Lower
Upper
Z-Score
60
40
20
0
0 1 2 3 4 5 6 7 8 9 10
90 91 92 93 94 95 96 97 98 99 100
Percentile
Z-score Distribution chr17
Z-Score (outliers = FALSE)
0.3
0.2
0.1
0.0
-0.1
Normal
TNBC
Phenotype
Lower
Upper
Z-Score
50
40
30
20
10
0
0 1 2 3 4 5 6 7 8 9 10
90 91 92 93 94 95 96 97 98 99 100
Percentile
Z-score Distribution chr18
Z-Score (outliers = FALSE)
0.4
0.2
0.0
Normal
TNBC
Phenotype
Lower
Upper
Z-Score
40
30
20
10
0
0 1 2 3 4 5 6 7 8 9 10
90 91 92 93 94 95 96 97 98 99 100
Percentile
phenotype
Normal
TNBC

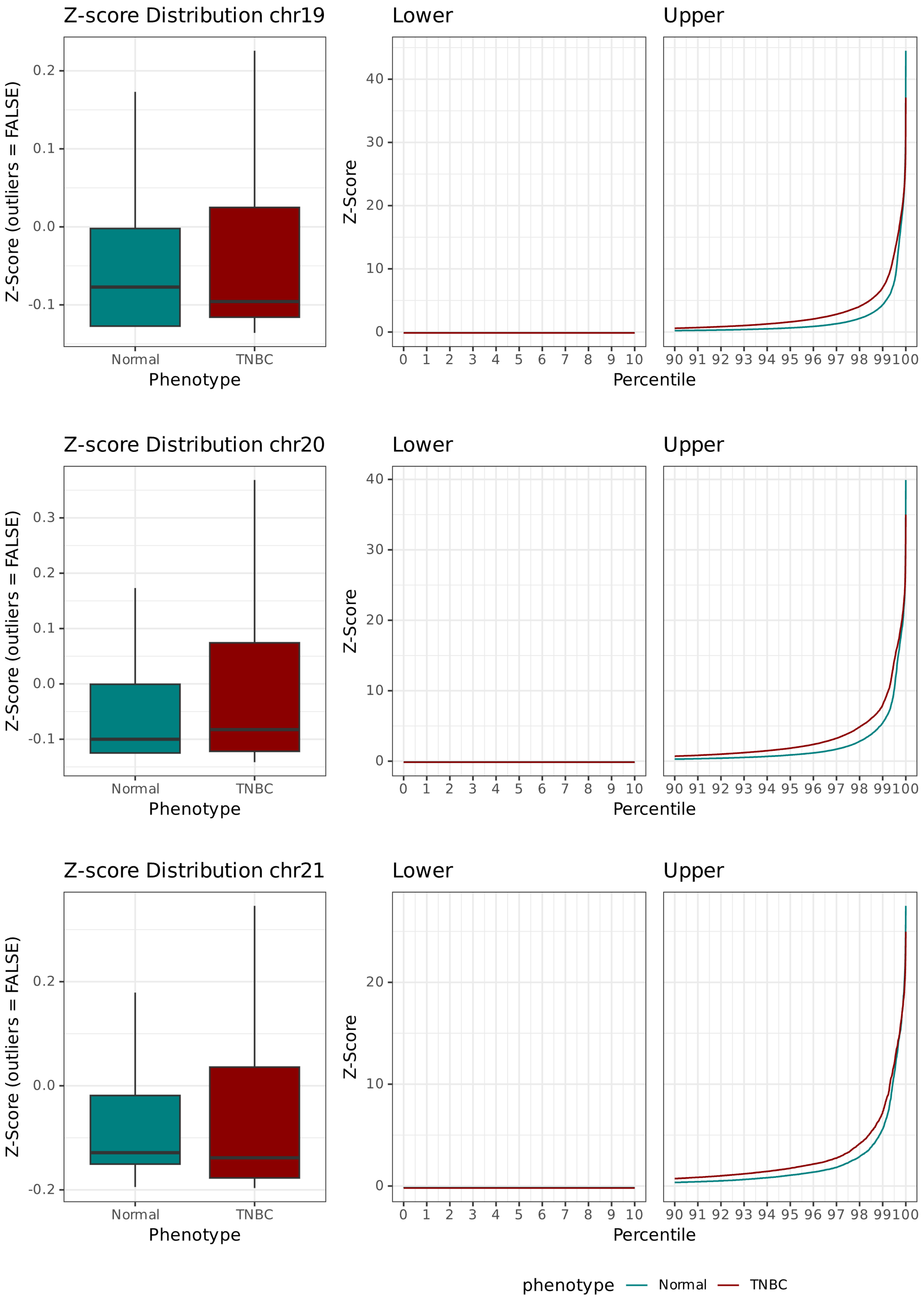

Z-score Distribution chr19
Z-Score (outliers = FALSE)
0.2
0.1
0.0
-0.1
Normal
TNBC
Phenotype
Lower
Upper
Z-Score
40
30
20
10
0
0 1 2 3 4 5 6 7 8 9 10
90 91 92 93 94 95 96 97 98 99 100
Percentile
Z-score Distribution chr20
Z-Score (outliers = FALSE)
0.3
0.2
0.1
0.0
-0.1
Normal
TNBC
Phenotype
Lower
Upper
Z-Score
40
30
20
10
0
0 1 2 3 4 5 6 7 8 9 10
90 91 92 93 94 95 96 97 98 99 100
Percentile
Z-score Distribution chr21
Z-Score (outliers = FALSE)
0.2
0.0
-0.2
Normal
TNBC
Phenotype
Lower
Upper
Z-Score
20
10
0
0 1 2 3 4 5 6 7 8 9 10
90 91 92 93 94 95 96 97 98 99 100
Percentile
phenotype
Normal
TNBC

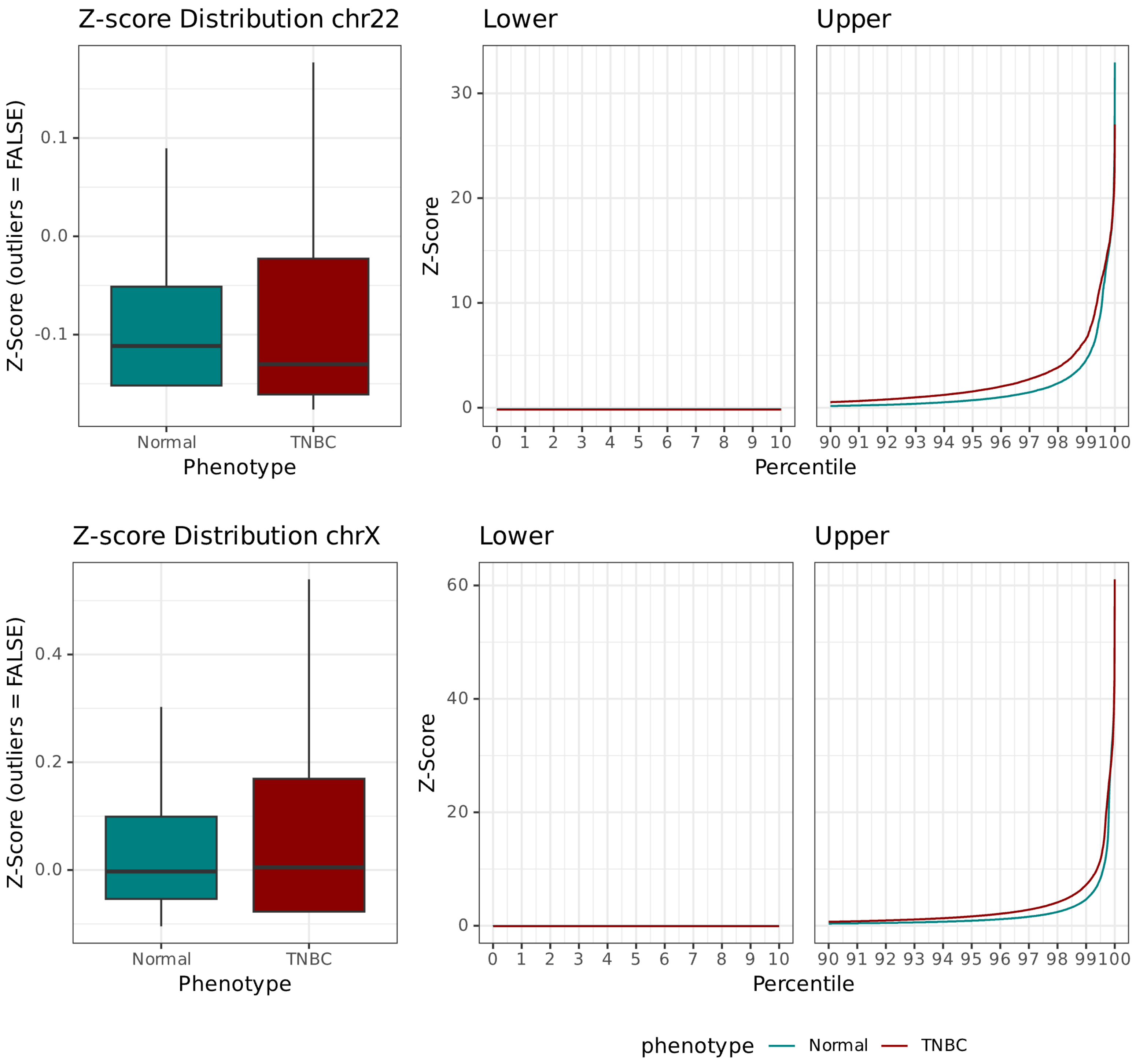

Z-score Distribution chr22
Z-Score (outliers = FALSE)
0.1
0.0
-0.1
Normal
TNBC
Phenotype
Lower
Upper
Z-Score
30
20
10
0
0 1 2 3 4 5 6 7 8 9 10
90 91 92 93 94 95 96 97 98 99 100
Percentile
Z-score Distribution chrX
Z-Score (outliers = FALSE)
0.4
0.2
0.0
Normal
TNBC
Phenotype
Lower
Upper
Z-Score
60
40
20
0
0 1 2 3 4 5 6 7 8 9 10
90 91 92 93 94 95 96 97 98 99 100
Percentile
phenotype
Normal
TNBC

SUPPLEMENTARY FIGURE 8

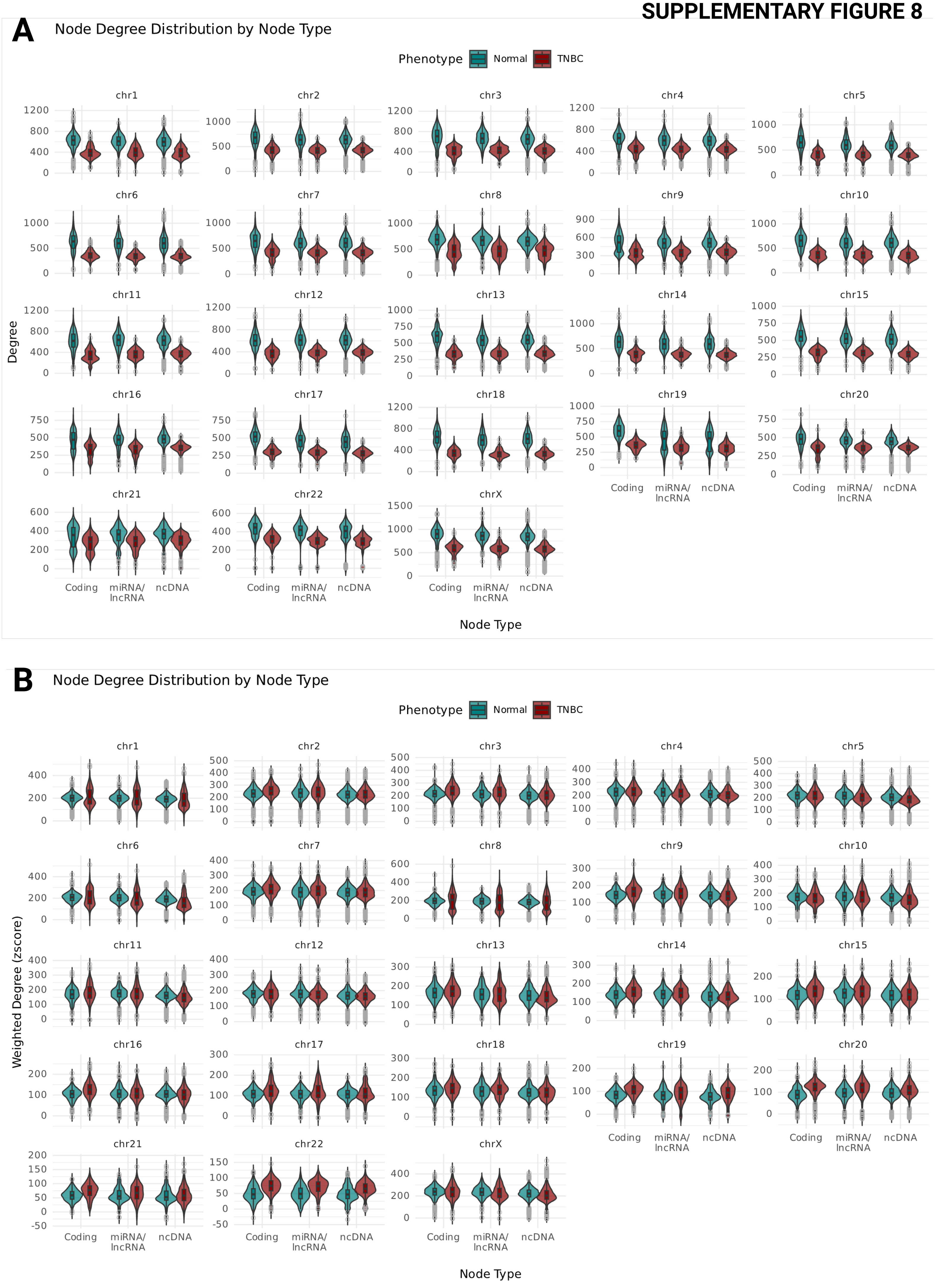

SUPPLEMENTARY FIGURE 9

A

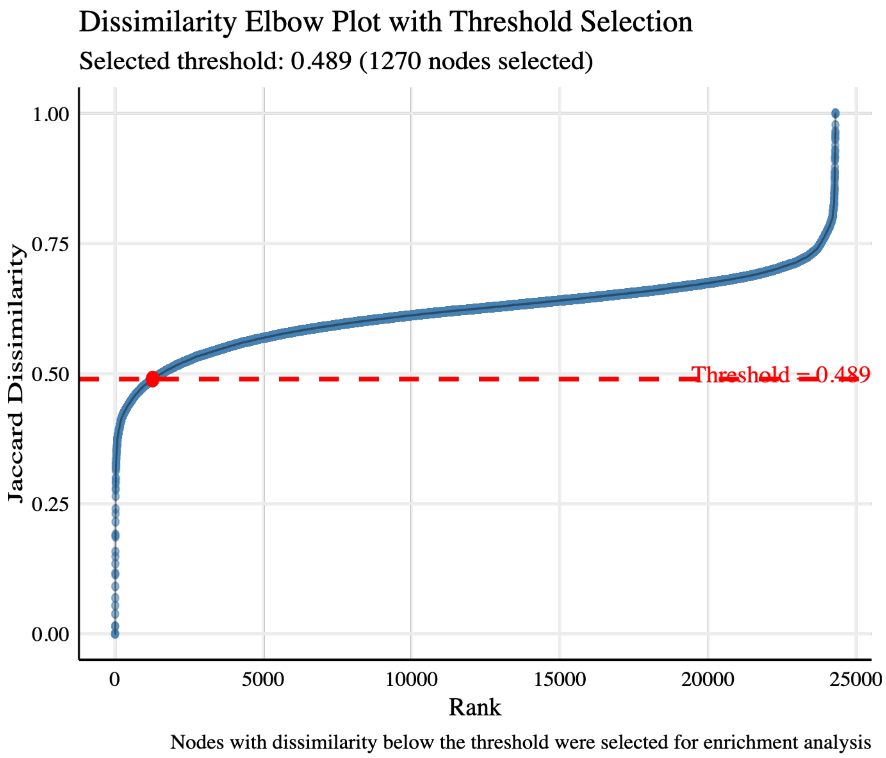

B

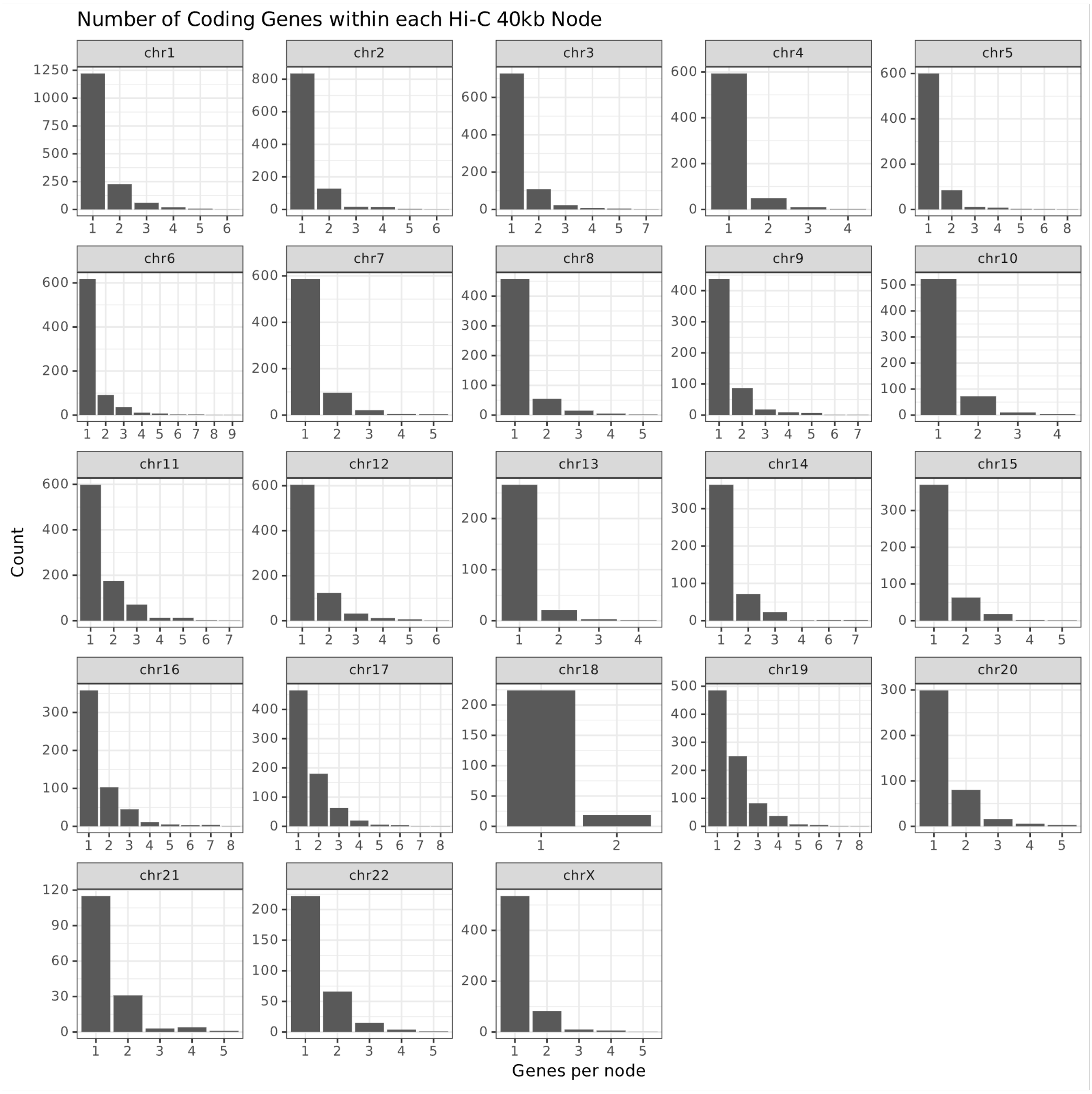

Supplement: Supplementary file 1 [file DataSheet1.zip › Supplementary_materials/SUPPLEMENTAL_FIGURES_0609.pdf]
