# Supplementary material for: Integration of chromosome conformation and gene expression networks reveals regulatory mechanisms in triple negative breast cancer
Source: Front Cell Dev Biol. 2025 Jul 4;13:1597245. doi: 10.3389/fcell.2025.1597245 (PMC12271745; doi:10.3389/fcell.2025.1597245)
Supplement: Supplementary file 1 [file DataSheet1.zip › Supplementary_materials/SUPPLEMENTAL_FIGURES_LEGENDS_0609.docx]

**Supplementary Figure 1**: Characteristics of the Hi-C Networks. (A) Pairwise Pearson Correlation of raw Hi-C matrices from the 3 Normal contralateral breast samples. (B) Pairwise Pearson Correlation of raw Hi-C matrices from the 3 TNBC contralateral breast samples. (C) Bar plot of the number of nodes comprising each chromosome’s Normal and TNBC Hi-C networks. At this 40kb Hi-C resolution each node corresponds to a genomic region that on average contains the TSS of one coding gene (orange), one noncoding RNA “miRNA/lncRNA” feature (green), or no genomic feature (blue) “ncDNA”. (D) Number of edges connecting the nodes of each chromosome’s Normal (green) and TNBC (dark red) Hi-C networks grouped by edge type. The type of edge is determined according to the feature content of the two nodes it connects.

**Supplementary Figure 2**: Genome Coverage by Hi-C Network Nodes. (A) Left column: The number of coding genes contained within the nodes of both Normal and TNBC Hi-C Networks by chromosome. Right column: The total number of coding genes annotated (gencode) for the GRCh38 reference genome by chromosome. (B) Ideogram of the GRCh38 human genome reference. Each chromosome is represented as a horizontal line with its g banding pattern and centromere region indicated in red. Below each chromosome a green (or dark red) track corresponds to the regions covered by the Normal (or TNBC) Hi-C network nodes. Above each chromosome a blue density plot indicates the gene density of the total coding genes annotated for the GRCh38 reference genome.

**Supplementary Figure 3:** (A-C) Whole Genome Heatmaps of Hi-C matrices at 500kb resolution, (A) Normal, (B) TNBC, (C) Difference between TNBC and Normal as log2 ratio. (D-F) Chromosome 10 Heatmaps of Hi-C matrices at 40kb resolution (D) Normal, (E) TNBC, (F) Difference between TNBC and Normal as log2 ratio.

**Supplementary Figure 4**: Genomic Distance of Hi-C Interactions by Edge Type. (A) Edge genomic distance distribution by edge (chromatin interaction) type. The density plot for the genomic distance values of each chromosome’s Normal and TNBC networks is drawn according to the interaction type: “C-C” Interactions between nodes containing coding gene(s), “C-R” Interactions between a node containing coding gene(s) and a node containing noncoding RNA gene(s), and “C-N” Interactions between a node containing coding gene(s) and a node containing no feature (noncoding DNA).

**Supplementary Figure 5**: Hi-C distance decay plots per chromosome. Each plot shows corrected contact frequency (y-axis) versus genomic linear distance (x-axis).

**Supplementary Figure 6**: (A-B) Chromosome X chord diagram with the node containing AR (Androgen Receptor) as the point-of-view (blue). The chromosome’s coordinates increase clockwise, and the first base pair position is at the top. AR’s top 200 (Z-score) intrachromosomal interactions with other nodes in normal breast tissue (A) and TNBC (B) are drawn. Edge color reflects Hi-C interaction Z-score value. The genes within each node are labeled. (C) Z-score distribution between Normal and TNBC.

**Supplementary Figure 7**: Hi-C count Z-Score. For each chromosome, left panel: boxplots of the chromatin interaction Z-Score values in the Normal (green) and TNBC (dark red) networks. The distributions are plotted excluding outliers. Middle panel: lower percentiles (0-10) of the Normal (green) and TNBC (dark red) edge Z-score distributions. Right panel: upper percentiles (90-100) of the Normal (green) and TNBC (dark red) edge Z-score distributions.

**Supplementary Figure 8**: Hi-C Networks Centrality Measures. (A) Node degree distribution. The distributions for each chromosome’s Normal (green) and TNBC (dark red) networks are shown side by side grouped by node type (Coding, miRNA/lncRNA, ncDNA). (B) Node weighted degree distribution. Node degree is weighed by its edges’ interaction count z-score. Distributions for Normal (green) and TNBC (dark red) networks are shown side by side grouped by node type (Coding, miRNA/lncRNA, ncDNA) for each chromosome.

**Supplementary Figure 9**: Coding Genes within Hi-C Network Nodes. (A) Bar plot of the number of genes within each node in the Hi-C intrachromosomal networks. The regions represented by nodes are Hi-C bins at a resolution of 40kb. (B) Dissimilarity Index Elbow Plot, the points represent Hi-C Network nodes with the X-axis showing their rank according to their Dissimilarity Index value and the Y-axis showing their Dissimilarity Value. The dashed red line marks the Jaccard dissimilarity threshold value used for enrichment analysis.
